# The effect of hyperkalemia and long inter-dialytic interval on morbidity and mortality in patients receiving hemodialysis: a systematic review

**DOI:** 10.1080/0886022X.2020.1871012

**Published:** 2021-01-21

**Authors:** Danai Bem, Daniel Sugrue, Ben Wilding, Ina Zile, Karin Butler, David Booth, Eskinder Tafesse, Phil McEwan

**Affiliations:** a Health Economics and Outcomes Research Ltd, Birmingham, UK; b Health Economics and Outcomes Research Ltd, Cardiff, UK; c AstraZeneca, Gaithersburg, MD, USA; d Swansea University, Swansea, UK

**Keywords:** Hyperkalemia, renal disease, hemodialysis, long inter-dialytic interval, systematic review

## Abstract

**Background:**

Patients with chronic kidney disease, especially those receiving hemodialysis (HD), are at risk of hyperkalemia (HK). This systematic review aimed to evaluate the prevalence of HK in patients with renal disease receiving HD and collate evidence on the effect of HK and differing HD patterns (i.e., long vs. short inter-dialytic intervals [LIDI and SIDI, respectively] in a thrice weekly schedule) on mortality.

**Methods:**

Comprehensive searches were conducted across six databases and selected conference proceedings by two independent reviewers up to September 2020. A hundred and two studies reporting frequency of HK, mortality, or cardiovascular (CV) outcomes in adult patients with acute, chronic or end-stage renal disease in receipt of HD were included. Narrative synthesis of results was undertaken with key findings presented in tables and figures.

**Results:**

Median prevalence of HK in patients with renal disease receiving HD was 21.6% and increased in patients receiving concomitant medications – mainly renin–angiotensin–aldosterone system inhibitors and potassium-sparing diuretics. Associations between elevated potassium levels and increased risk of both all-cause and CV mortality in the HD population were consistent across the included studies. In addition, there was a rise in all-cause and CV mortality on the day following LIDI compared with the day after the two SIDIs in patients on HD.

**Conclusions:**

Evidence identified in this systematic review indicates a relationship between HK and LIDI with mortality in patients with renal disease receiving HD, emphasizing the need for effective monitoring and management to control potassium levels both in emergency and chronic HD settings.

## Introduction

Hyperkalemia (HK) is a clinically important electrolyte abnormality characterized by an elevated serum potassium (S-K^+^) concentration above the normal range of 3.5–5.0 mmol/L [1]. While there is no universally agreed definition for HK, the most widely used thresholds for the categorization of mild, moderate and severe HK are 5.5–5.9, 6.0–6.4 and ≥6.5 mmol/L, respectively [2,3]. Mild HK may be associated with symptoms such as nausea, fatigue or muscle weakness [2]; however, more severe HK can cause alterations in cardiac physiology resulting in chest pain, cardiac dysrhythmia, shortness of breath, and – in very severe cases – cardiac arrest and death [4,5].

Renal excretion is the main route of K^+^ elimination; therefore, renally impaired patients are at increased risk of HK [6]. Indeed, HK is reported in approximately 50% of patients with chronic kidney disease (CKD) [7,8], with prevalence increasing alongside disease severity. Patients with CKD that progress to end-stage renal disease (ESRD) or patients with acute renal failure are initiated and often maintained on hemodialysis (HD). HK is common in the dialysis setting [9]; however, its true prevalence in patients receiving dialysis is unknown.

HD is normally prescribed thrice weekly with schedules of Monday-Wednesday-Friday (MWF) or Tuesday-Thursday-Saturday (TTS). The two shorter (48 h) breaks between HD sessions are referred to as the short inter-dialytic interval (SIDI) and the extended (72 h) break is known as the long inter-dialytic interval (LIDI). Given the impaired ability of patients receiving HD to excrete K^+^ and other electrolytes and their high prevalence of cardiovascular (CV) disease, patients are at increased risk of hospitalizations, cardiac arrhythmias and mortality following LIDI [10,11]. However, the interplay between S-K^+^, LIDI and clinical outcomes remains uncertain.

The aim of this study was to undertake a systematic literature review (SLR) to evaluate the effect of HK and LIDI on mortality in patients with renal disease or acute renal failure receiving HD. The findings will have the potential to provide further information on the frequency and management of HK to support healthcare professionals working in the dialysis setting.

## Methods

This systematic review was conducted and reported according to the Preferred Reporting Items for Systematic Reviews and Meta-Analyses (PRISMA) statement [12] with the protocol developed *a priori*.

### Search strategy

Comprehensive searches of electronic databases (MEDLINE, MEDLINE In-Process, Embase, Cochrane Library, University of York Center for Reviews and Dissemination) were conducted from database inception to 1st September 2020. A sample search strategy is provided in Supplementary Table S1. In addition, conference proceedings (American Society of Nephrology, European Renal Association-European Dialysis and Transplant Association, and International Society of Nephrology) were reviewed for the past four years for any studies that may not have been captured from database searches, and bibliographies of relevant studies and systematic reviews were visually scanned to identify relevant primary studies.

### Study selection

EndNote X9 was used to facilitate the removal of duplicate records, study selection and referencing of literature search results. Two reviewers independently screened articles for eligibility using pre-determined inclusion/exclusion criteria according to a population- intervention-comparator-outcome-study design (PICOS) framework (Table 1). Any discrepancies between reviewers were resolved by consensus or involvement of a third reviewer.

**Table 1.** Eligibility criteria for the identification of studies describing HK and LIDI in HD patients.

PICOS element	Inclusion criteria	Exclusion criteria
Population	Adults (aged ≥ 18 years) with acute renal failure or chronic/end-stage renal disease in receipt of hemodialysis	Patients not receiving hemodialysis Paediatric patients (aged < 18 years) Patients without acute renal failure or chronic/end-stage renal disease
Intervention and comparison	Hemodialysis	Interventions other than hemodialysis Other dialysis modalities (e.g., peritoneal dialysis, home dialysis)
Outcomes	Prevalence, incidence of HK All-cause mortality Cardiovascular mortality Cardiovascular events (including MI, stroke, heart failure, cardiac arrhythmias, TIA, angina)	Outcomes of interest not reported
Study design	Any relevant study design (including RCTs, non-RCTs, epidemiological studies, observational studies)	Case series, case reports Guidelines, letter, editorial, review, retracted Systematic reviews^a^ Non-human studies
Language restriction	English language only	Studies published in languages other than English
Publication date restriction	None	None

HK: hyperkalemia; MI: myocardial infraction; RCTs: randomized controlled trials; TIA: transient ischemic attack.

^a^Such study designs were used only to identify potentially relevant primary studies that had not been captured through database searches.

### Data extraction and quality assessment

Relevant information on study characteristics, cohort details and study outcomes were extracted from all included studies by a single reviewer and quality-checked by a second reviewer for accuracy and completeness. All data were extracted in a consistent manner using a standardized data extraction form.

Quality assessment of observational studies was performed using a checklist modified from the Downs and Black instrument [13]. The Cochrane risk of bias tool was used to assess methodological quality and reporting in randomized controlled trials (RCTs) [14].

### Data synthesis

Narrative synthesis of included studies was undertaken. Between study heterogeneity and limited reference to the timepoint, where mortality was reported, precluded meta-analysis. Simple analysis was performed in the statistical package R [15] with summary statistics presented as mean (± standard deviation) for demographic data, median (interquartile range) for prevalence and median (range) for mortality data.

Studies reporting a specific threshold for defining HK were included in the review and the HK definition was recorded as such. When studies did not provide a HK definition then they were included in the review if they reported the proportion of patients with pre-dialysis S-K^+^ concentrations ≥5.0 mmol/L. Prevalence was defined as the proportion of patients with HK in the total HD population included in a study in a given period.

A standardized definition for the different inter-dialytic intervals was used; for studies where results were presented based on days of the week (i.e., MWF or TTS HD schedule), data were aligned to SIDI and LIDI periods to enable comparison. Thus, Mondays (MWF schedule) and Tuesdays (TTS schedule) were categorized as ‘day after LIDI’, Wednesdays (MWF schedule) and Thursdays (TTS schedule) as ‘day after SIDI1’, and Fridays (MWF schedule) and Saturdays (TTS schedule) as ‘day after SIDI2’. An ‘end of LIDI’ group was used to refer to Sundays (MWF schedule) or Mondays (TTS schedule).

## Results

**Figure 1.**
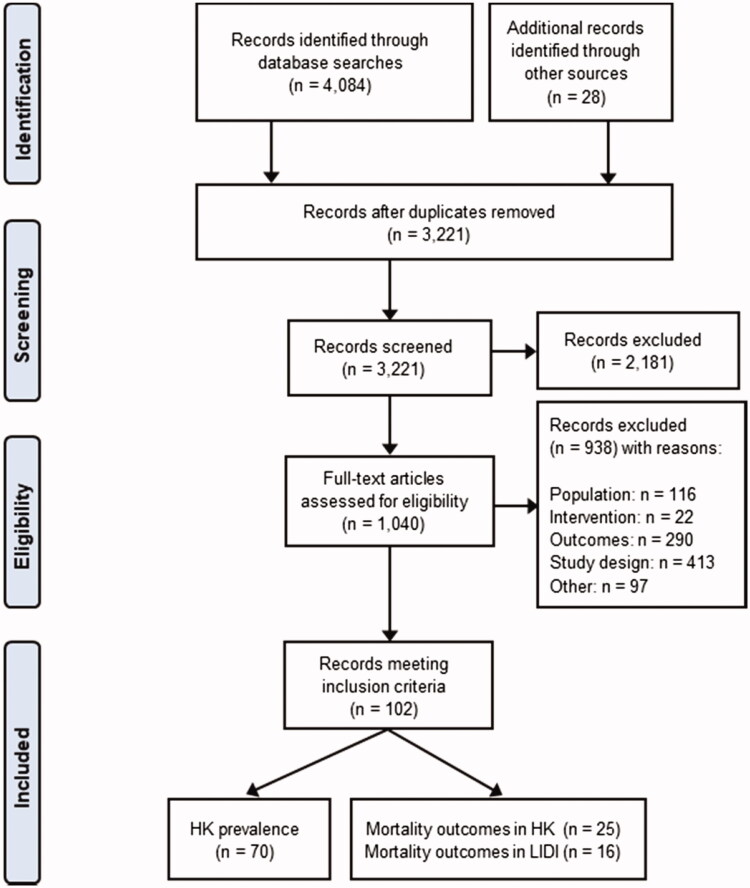
PRISMA flow diagram illustrating the study selection process. HK: hyperkalemia; LIDI: long inter-dialytic interval.

Systematic searches yielded 3221 records after the removal of duplicates, with 102 studies included in the final synthesis. The study selection process is illustrated in a PRISMA flow diagram [12] (Figure 1).

### HK in patients with CKD or acute renal failure receiving HD

Seventy studies reported on the frequency of HK [16–85], 25 studies reported on the associated mortality [21,22,34,41,44,45,48,68,70,86–101] in HD patients with CKD or acute renal failure, and nine studies [21,22,34,41,44,45,48,68,70] reported on both frequency of HK and associated mortality.

#### Prevalence of HK in patients receiving HD

Of the 70 studies reporting on the frequency of HK (Supplementary Table S2), 45 were observational studies, 15 were RCTs, four were nonrandomized trials and six did not explicitly report a study design. The population size varied significantly between studies ranging from 5–74,219 patients. The mean age of patients was 58.3 ± 6.4 years and 59% were male. Of the identified studies, 40 examined patients with ESRD, 21 included CKD patients (not explicitly categorized as ESRD patients) in receipt of HD, four included patients with acute kidney injury (AKI) and five focused on emergency HD for the treatment of renal failure emergencies (Table 2).

**Table 2.** Summary of patient characteristics and outcomes in studies reporting frequency of HK in HD patients stratified by population.

HD population (no. of studies)	Publication type	Study design	Cohort size (N)	Age (years)^a^, % male	HD vintage (months)^a,b^	Median prevalence (IQR)	Incidence rate
ESRD (*n* = 40) [9,17,20–23,25,26,29–31, 35–41,43,44,46,47, 50,54,55,59,63, 65–67,70,72,74–79, 82,84,85]	33 Full texts 7 Abstracts	22 Observational 3 non-RCT trials 12 RCTs 3 NR	10–55,183	60.6 (5.3), 59%	42.3 (27.9) 3x weekly schedule	19.0% (7.8–38.9%)	15.1–26.0 events per 100 patient-months
CKD (*n* = 21) [18,19,24,27,32–34, 42,45,48,49, 51,53,56–58,60,61, 64,69,71]	17 Full texts 4 Abstracts	14 Observational 2 non-RCT trials 3 RCTs 2 NR	5–74,219	56.3 (6.9), 58.8%	59.8 (20.1) 3x weekly schedule	24.0% (7.6–38.8%)	–
AKI (*n* = 4) [28,52,73,80]	2 Full texts 2 Abstracts	All observational	46–105	52.8 (14.7), 63.8%	Intermittent HD	27.3% (16.0–38.4%)	–
eHD (*n* = 5) [16,62,68,81,83]	3 Full text 2 Abstract	4 Observational 1 NR	37–489	53.8 (7.5), 60.2%	eHD	24.0% (21.1–37.6%)	–

AKI: acute kidney injury; CKD: chronic kidney disease; eHD: emergency hemodialysis; ESRD: end-stage renal disease; HD: hemodialysis; IQR: interquartile range; NR: not reported; RCTs: randomized controlled trials.

^a^Mean (SD).

^b^Reported in a limited number of studies only.

Forty-eight studies did not consider any medication when reporting outcomes of interest, whereas 22 studies stratified results by the co-administered drug, including blood pressure medication (renin-angiotensin-aldosterone system inhibitors [RAASi]), K^+^-sparing diuretics (spironolactone [SPL], eplerenone), K^+^-binding agents (sodium polystyrene sulfonate [SPS], patiromer [PAT], sodium zirconium cyclosilicate [SZC]) and drugs for the treatment of hyperparathyroidism and anemia among others. Diabetes (*n* = 32) and hypertension (*n* = 18) were the most frequently reported comorbidities in the included HD populations (42% with diabetes and 76.2% with hypertension on average), followed by heart failure (31%) and coronary artery disease (35.7%).

**Figure 2.**
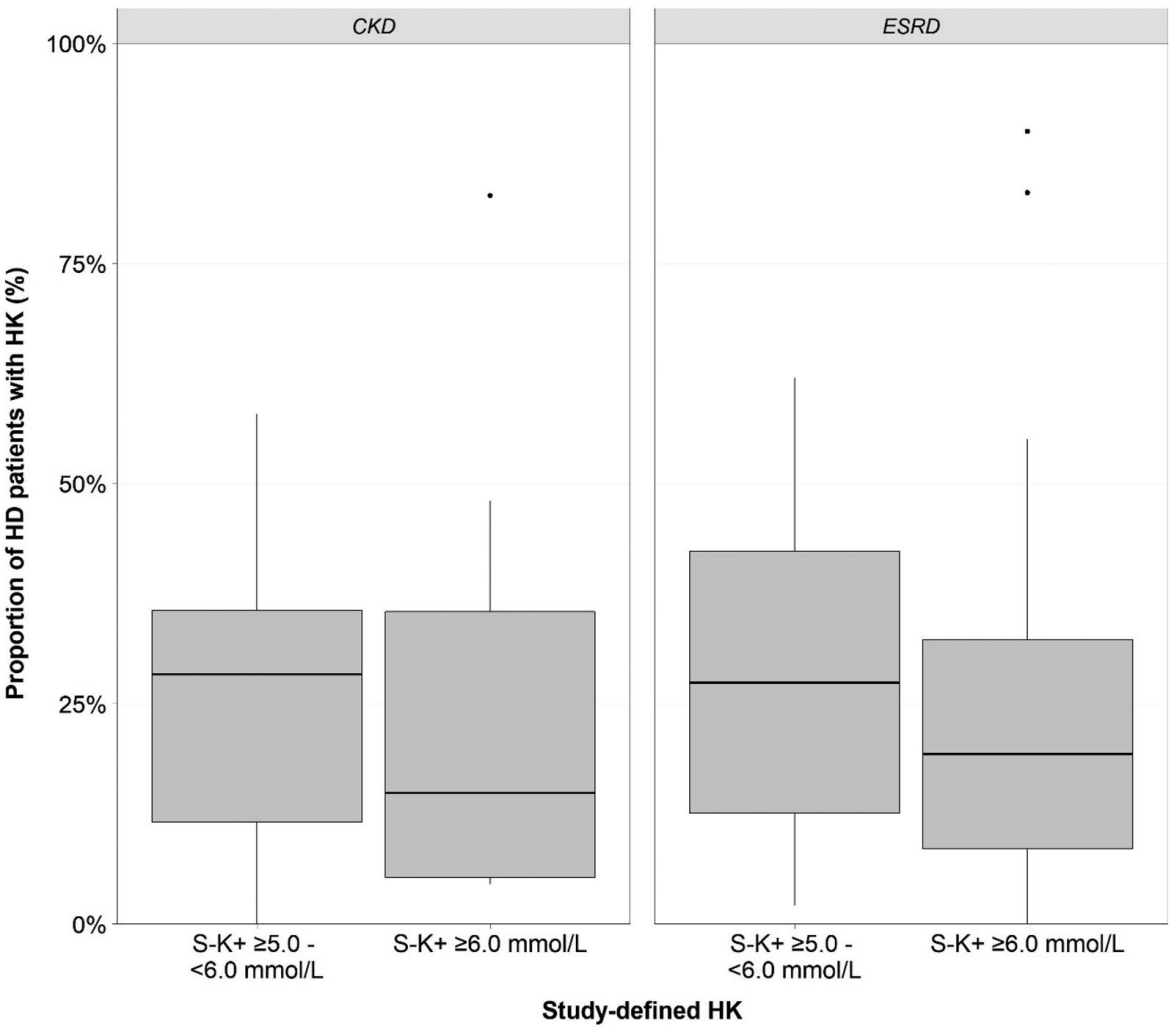
Median prevalence of HK (%) across the 70 included studies stratified by study-defined S-K^+^ thresholds. CKD: chronic kidney disease; ESRD: end-stage renal disease; HD: hemodialysis; HK: hyperkalemia; S-K^+^: serum potassium.

The definition of HK varied between studies. Where reported (*n* = 46), the majority of studies defined HK as pre-dialysis S-K^+^ concentration of ≥5.5 mmol/L (54%) and to a lesser extent ≥5.0 mmol/L (15%) and ≥6.0 mmol/L (22%); the rest of the studies reported moderate to severe HK without providing a HK threshold. Overall, 67 studies [16–21,23–45,47–69,71–85] reported the prevalence of HK as the proportion of patients with HK in the HD population studied and four reported HK incidence rates [22,46,70,82]. Median HK prevalence in HD patients with renal disease was 21.6% (7.3–39.3%) and was comparable between ESRD and CKD patients (Table 2). The prevalence of study-defined HK, when using different S-K^+^ thresholds, is presented in Figure 2. Median prevalence of milder HK in patients with S-K^+^ 5.0–6.0 mmol/L (excluding all cases of serious HK, i.e., ≥6.0 mmol/L, where possible) was 27.4% (12.1–36.9%) and median prevalence of more serious HK (S-K^+^ ≥6.0 mmol/L) was 19.3% (7.3–35.4%). In patients with AKI, HK was the most common indication for initiation of HD in 27.3% of the cases, and HK was observed in 24% of patients requiring emergency HD (Table 2).

Thirteen studies [19–21,23,35,36,42,43,54,57,64,66,67,76] investigated the impact of drugs, mainly RAASi and K^+^-sparing diuretics, on the occurrence of HK. An increase in the proportion of HD patients presented with HK was observed in patients receiving medication on top of HD (24.4%; 7.3–46.1%) compared with those that received no medication or placebo (12.0%; 2.1–18.6%).

#### Mortality in HD patients with HK

Mortality outcomes were reported in 25 studies [21,22,34,41,44,45,48,68,70,86–88,90–102] (Supplementary Table S3). Studies were mostly observational and therefore limitations such as confounding by indication should be considered when interpreting these results; only one study [22] was an RCT. The size of the enrolled population varied significantly between studies (range: 8–51,297 patients), the mean age of patients was 60.1 ± 4.1 years and 54% were male. Sixteen studies enrolled patients with ESRD, eight with CKD (not explicitly categorized as ESRD patients) in receipt of HD and one study included patients receiving emergency HD due to renal failure.

Studies varied in the outcome measure used to report mortality. Twelve studies [22,87,88,91–99] reported the proportion of patients that died due to HK in the overall HD population ranging from 0–12.5% (follow-up range: 3–66 months, where reported) (Table 3).

**Table 3.** Studies reporting mortality due to HK in patients receiving HD (*n* = 12).

Author, year, country	Population	Study period	Subgroup (N)	Age (years)^a^, % male	S-K^+^ levels (mmol/L)	Follow-up (months)	Death due to HK
Balle et al. 1990, Germany	ESRF due to analgesic-associated nephropathy	1970–1986	Total population (55)	61 (44–79), 34.5%	NR	57	7.3%
Chaaban et al. 2013, United Arab Emirates	CKD	NR	Control group (30)	48.9 (18.7), 52%	NR	3	0%
Charytan et al. 2017, USA	ESRD	NR	Placebo (51)	NR	> 6.0	8.3	0%
Jadoul et al. 2012, International	ESRD	1996–2008	Total population (37,765)	NR	> 5.0	19.1^a^	1.3%
Li et al. 2012, China	ESRD	2006–2011	Total population (268)	NR	NR	60	4.5%
Li et al. 2020, China	CKD	2011–2015	Total population (210)	56.6 (16.6), 59.5%	NR	49.8^b^	4.4%
Lomonte et al. 2004, Italy	ESRD/Leprosy	1980–2003	Total population (8)	61 (8.9), 75%	NR	NR	12.5%
Morduchowicz et al. 1992, Israel	ESRF	NR	Total population (84)	NR	NR	66	5%
Onuigbo et al. 2013, Italy	ESRD	2007–2010	Total population (466)	65.6 (19–97)^b^, 49.1%	NR	28.9^a^	3%
Poulikakos et al. 2015, UK	Renal failure	NR	Total population (75)	60 (14), 68%	NR	35.9^a^	1.3%
Pun et al. 2012, USA	ESRD	2002–2005	Total population (363)	69 (59–78)^b^, 55.4%	NR	NR	1.3%
Shibata et al. 1983, Japan	Renal failure	NR	Total population (62)	50 (26–78), 67.7%	NR	NR	3.2%

CKD: chronic kidney disease; ESRD: end-stage renal disease; ESRF: end-stage renal failure; HK: hyperkalemia; NR: not reported; S-K^+^: serum potassium.

^a^Mean (SD) or mean (range).

^b^Median (IQR).

Eight studies [41,45,48,68,86,100–102] reported percentage all-cause mortality in different groups of patients. Alagusundaramoorthy et al. [86] undertook a chart review of 346 adult ESRD patients admitted to hospital, who had HD for severe HK, and reported that in-hospital mortality for these patients was 6.9%. Chatoth et al. [102] examined outcomes of patients with moderate to severe HK undergoing HD in a large ESRD provider network in the USA. For S-K^+^ >6.0 mmol/L, all-cause mortality over a 6-month period was 0.0%, 1.8% and 2.5% for patients receiving K^+^-binding agents PAT and SPS, and no K^+^-binder, respectively, compared to 2.8%, 2.5%, and 2.3% for S-K^+^ >5.5 mmol/L. Hwang et al. [41] presented data from a retrospective single center study of ESRD patients receiving HD. Patients were divided into three groups according to the last mid-week pre-dialysis S-K^+^ concentrations: hypokalemia (<3.5 mmol/L), normokalemia (3.5–5.5 mmol/L), and HK (>5.5 mmol/L). The maximum duration of the follow-up period was 4.5 years. All-cause mortality was lowest for patients with normokalemia at 18.7%, compared to 21.6% for patients with HK. Kim et al. [45] examined the differences in the relationship between S-K^+^ and all-cause mortality across non-Hispanic whites, African-Americans and Hispanics in a contemporary cohort of over 100,000 incident HD patients, who had a median follow-up of 1.3 years. The data demonstrated that while mortality was increased for whites and African–Americans with higher S-K^+^, this was not observed in Hispanic HD patients; all-cause mortality was 35.4%, 27.5% and 15% for white, African-American and Hispanic HD patients respectively with S-K^+^ >5.5 mmol/L, compared with 32.6%, 20.6% and 16.2% for patients with S-K^+^ 5.0–5.5 mmol/L. Kovesdy et al. [48] reported results from a multicenter cohort of 81,013 CKD patients on maintenance HD (mean follow-up of 36 months). The study findings suggested that S-K^+^ between 4.0 and 5.3 mmol/L was associated with the lowest death rate (28–30%), whereas S-K^+^ >5.6 mmol/L was associated with increased all-cause mortality (≥32%). Yalin et al. [68] reported data from 177 adult patients who underwent emergency HD between 2000 and 2010 in which all-cause mortality rate was 41.8%. Zulham et al. [101] observed that early death in diabetic ESRD patients on HD was 49% in patients with HK compared with 25.2% in these without HK. Finally, Trajceska et al. [100] reported that 19.5% of ESRD patients with HK died during the 36 months follow-up period.

Hazard ratios (HRs) for mortality were reported in five studies [21,34,44,70,90]. For most studies, HR for all-cause mortality was increased with higher S-K^+^ levels, although the differences were not significant (Supplementary Table S3).

Four studies [34,41,45,48] reported CV mortality. CV mortality was generally increased in the HK population (>5.5 mmol/L) compared to patients with normokalemia (3.5–5.5 mmol/L); 9.8% compared to 5.5% in Hwang et al. [41], 13.0–14.0% compared to 10.0–11.0% in Kovesdy et al. [48], and 6.1–13.0% compared to 7.0–11.3% in Kim et al. [45]. Genovesi et al. [34] reported 3-year cumulative incidence of deaths in a cohort of 476 patients; for patients without HK (S-K^+^ <6.0 mmol/L) the 3-year cumulative incidence of deaths was 12.6% compared to 18.2% for patients with HK (≥6.0 mmol/L). An increase in cardiac arrest, coronary artery disease (CAD) and cardiac arrhythmia was also observed in patients with HK compared with normal S-K^+^ populations, with risk increasing with HK severity [41,44].

### Mortality associated with the inter-dialytic period

Sixteen studies [10,34,103–117] were identified reporting mortality outcomes at different days of the week based on the HD schedule (Supplementary Table S4). Using a standardized definition for the different inter-dialytic intervals, the following groups were recorded: day after LIDI; day after SIDI1; day after SIDI2; end of LIDI; during LIDI; during SIDI.

The majority of studies were observational, with the cohort size ranging from 28–61,152 patients. The mean age of included patients, where reported, was 61.9 ± 4.8 years and 59% were male. Nine studies recruited patients with ESRD and seven with CKD (not explicitly categorized as ESRD patients) receiving HD. All included studies, apart from one [109], reported a thrice weekly HD schedule.

Five studies [10,105,106,111,116] reported all-cause mortality. Three of the studies [10,105,106] reported all-cause mortality rates with all observing an increased mortality on the day after LIDI compared with the day after the short intervals (22.1 vs. 18.9, 8.4 vs. 5.2, and 17 vs. 14 deaths per 100 person-years, respectively); follow-up ranging from 18.5–60.0 months. Two of the studies [111,116] reported mortality as proportion of deaths in the overall study population stratified by MWF or TTS HD schedule. The data from these studies also demonstrated a higher all-cause mortality on the day after LIDI (median [range]: 19.7% [17.9–23.7%] for MWF, 19.2% [16.9–22.3%] for TTS) compared with the day after SIDI (median [range]: 14.8% [13.3–19.1%] for MWF, 15.7% [11.9–17.9%] for TTS) and end of LIDI (median [range]: 12.4% [12.1–15.7%] for MWF, 14.4% [10.7–15.7%] for TTS).

Four studies [34,103,104,115] reported the proportion of patient deaths recorded as sudden death at the end or after the LIDI period. Two studies defined sudden death as cardiac death. Bleyer et al. (1999) reported that in a cohort of 6137 ESRD patients receiving HD 18.1–20.8% of all deaths were sudden deaths occurring on the day after LIDI compared with 12.7–16.7% on the day after SIDI or at the end of LIDI [104], whereas Wong et al. reported that in a total of ten deaths in a cohort of 50 CKD patients followed-up for 18 months, eight were sudden deaths, all of which occurred during LIDI [115]. Two studies defined sudden death as unexpected death without known etiology occurring within one hour of the onset of symptoms. Genovesi et al. reported that in a cohort of 476 CKD patients 40.6% of all deaths were sudden deaths occurring during LIDI compared with 25–34.4% on the day after the two SIDI periods [34], whilst Bleyer et al. (2006) reported that from the 228 patient deaths reviewed 20.6% were sudden deaths occurring at the end of LIDI compared to 11.3% on the day after LIDI [103].

Five studies [10,104,108,109,116] reported CV mortality using different outcome measures. All showed increased CV mortality on the day after LIDI compared with the day after SIDI. In one study [108] of 10,338 ESRD patients the odds ratio (OR) associated with CV mortality on the day after LIDI was 1.27 (MWF schedule) and 1.10 (TWS schedule), compared with a range of 0.84–1.02 for other days during the HD schedule. In another study [116] conducted in the US population showed that the relative risk associated with CV mortality on the day after LIDI was 1.45 (MWF schedule) and 1.56 (TTS schedule), compared with the day after SIDI (range: 1.03–1.09) or end of LIDI (range: 0.94–1.16). Data for other countries included in this study followed a similar pattern, demonstrating that cardiac deaths were more likely to occur on the day after LIDI than other periods during the HD schedule. Three studies reported on the percentage of CV deaths in the overall study population; in a study of 6137 ESRD patients on HD CV mortality was 18.5–20.2% on the day after LIDI compared with 13.5–14.9% on the day after SIDI and 13.3–15.2% at the end of LIDI [104], in a study of 10,338 ESRD patients on HD CV mortality was 8.8–9.7% on the day after LIDI compared to 7.6–8.0% on the day after SIDI and 6.7% at the end of LIDI [108], and in a study of 240 ESRD patients on HD CV mortality was 12.9% on the day after LIDI compared to 6.3% on the day after SIDI [109]. One study reported CV mortality rates per 100 person-years and reported significantly higher mortality rates on the day after LIDI than on other days of the week (10.2 vs. 7.7–8.1 per 100 person-years, respectively) [10].

The most commonly encountered CV events in the LIDI period was cardiac arrhythmia [112–115] and cardiac arrest [10,107]. MI [10], atrial fibrillation [112], ventricular tachycardia [113] and cardiopulmonary resuscitation (CPR) [110] were also reported.

### Association between LIDI and HK

Three studies reported an association between LIDI and prevalence of HK (Table 4). Chevarria et al. [118] monitored 20 ESRD dialysis patients for 12 months and reported an association between LIDI and S-K^+^ >6.0 mmol/L (OR: 2.8 [95% CI, 1.47–5.33]). Similarly, Yusuf et al. [70] observed in four annual cohorts of HD patients ranging from 28,774 to 36,888 patients that prevalence of HK on the day after LIDI was 2.0 − 2.4 times higher than on the day after SIDI. Finally, Singh et al. [63] observed in 240 ESRD patients on HD that were monitored for 12 days that HK was more common during LIDI with 36 − 50% of patients having pre-dialysis HK after LIDI.

**Table 4.** Studies reporting an association between HK and the inter-dialytic period.

Author, year, country (publication type)	Population	S-K^+^ levels (mmol/L)	Subgroup (N)	Follow-up	HD schedule	HK prevalence	
						Outcome measure	Value (95% CI)
Chevarria et al. 2010, Spain (Abstract)	ESRD on HD	>6.0	Total population (20)	12 months^a^	3x weekly; MWF or TTS	OR	2.8 (1.47–5.33)
Singh et al. 2017, USA (Abstract)	ESRD on HD	>5.0	Dialysate K^+^ <3.0 mmol/L (179)	12 days	Unclear; 6 HD within 12 days	Percentage	50.0
			Dialysate K^+^ ≥3.0 mmol/L (33)	12 days			36.0
Yusuf et al. 2016, USA (Full text)	ESRD on maintenance HD	≥5.5	Day after LIDI 2007 cohort (28,774)	9 months^a^ (19,336^b^)	3x weekly; MWF or TTS	Rate per 100 patient-months	58.7 (57.7–59.8)^c^
			Day after LIDI 2008 cohort (34,788)	9 months^a^ (23,503^b^)			62.2 (61.2–63.2)^c^
			Day after LIDI 2009 cohort (34,571)	9 months^a^ (24,271^b^)			62.9 (61.9–63.9)^c^
			Day after LIDI 2010 cohort (36,888)	9 months^a^ (23,272^b^)			61.6 (60.6–62.6)^c^
			Day after SIDI 2007 cohort (28,774)	9 months^a^ (38,673^b^)			28.8 (28.3–29.3)^c^
			Day after SIDI 2008 cohort (34,788)	9 months^a^ (47,005^b^)			27.6 (27.2–28.1)^c^
			Day after SIDI 2009 cohort (34,571)	9 months^a^ (48,541^b^)			26.3 (25.8–26.7)^c^
			Day after SIDI 2010 cohort (36,888)	9 months^a^ (46,543^b^)			26.2 (25.8–26.7)^c^

CI: confidence interval; ESRD: end-stage renal disease; HD: hemodialysis; HK: hyperkalemia; LIDI: long inter-dialytic interval; MWF: Monday-Wednesday-Friday; OR: odds ratio; SIDI: short inter-dialytic interval; S-K^+^: serum potassium; TTS: Tuesday-Thursday-Saturday.

^a^Mean.

^b^Cumulative follow-up time in months.

^c^Rate of HK was computed as a ratio of total number of HK episodes and cumulative follow-up time; 95% CI for the rates of HK were calculated based on normal approximation.

### Quality assessment

**Figure 3.**
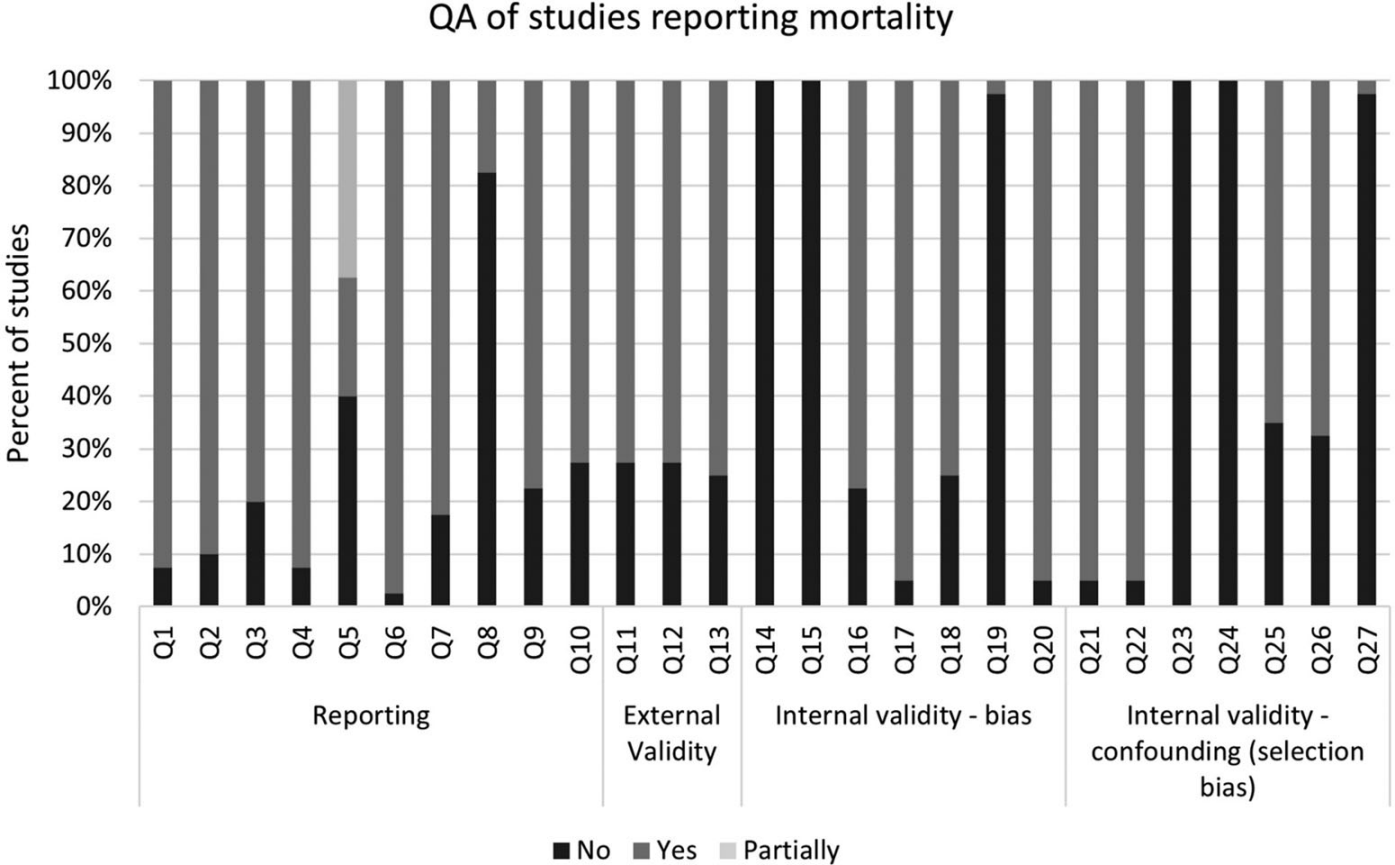
Quality assessment of the 40 observational studies reporting on mortality. One additional study was an RCT but published as a conference proceeding making the quality assessment not informative.

Quality assessment was performed for the 41 studies reporting mortality outcomes as this was the main objective of the review (Figure 3). Overall, methodological quality and reporting were adequate in most studies, except reporting of all adverse events; however, this is of lesser importance as we specifically regarded mortality in this analysis. External validity was generally adequate. Regarding internal validity, due to the observational study design of the included studies, blinding and randomization were absent. Patient compliance with the intervention was not well assessed. The other tool items were met in 60% to 97.5% of the studies. The only included RCT was reported as a conference proceeding, and the quality assessment was not informative.

## Discussion

In total, 102 studies met the eligibility criteria for inclusion in this systematic review. A median HK prevalence of 21.6% was observed in patients with renal disease receiving HD, based on study-defined criteria for HK, with more serious cases of HK occurring in an estimated 19.3% of the HD population. This is significantly higher than the prevalence in the general population which has been estimated to be 2–3% [119,120]. The incidence rate of HK in ESRD patients ranged from 15.1 to 26.0 events per 100 patient-months.

It is well established that HK is a major burden in terms of increased risk of CV outcomes (due to alterations in cardiac electrophysiology) and mortality. Death due to HK was observed in approximately 4% (range: 0–12.5%) of the overall HD population. Data identified in this review also indicated that both all-cause and CV mortality were increased in patients with HK receiving HD compared to those with normal S-K^+^ levels. These findings align with a recently conducted systematic review that also reported an increased risk of both all-cause and CV mortality with S-K^+^ levels outside the normokalemia range [121]. Thus, the evidence collated in this SLR supports the existence of an association between elevated S-K^+^ levels and mortality across patient populations with renal disease receiving HD. However, the heterogeneity observed in the included studies relating to patient characteristics, the intervals of S-K^+^ used to define HK, and the outcome measures and timepoints used to report mortality may impact on the generalizability of the review findings. It should be noted that the prevalence of comorbidities in CKD patients such as diabetes, hypertension and CV disorders alongside HK may potentiate any CV risk and death rates in the HD population. In addition, there are indications that HK may not only have effects on cardiac excitability but may also contribute to peripheral neuropathy and cause renal tubular acidosis, thus increasing the risk of death [122].

Current evidence indicates that the risk of mortality in patients receiving HD peaks the day after LIDI potentially due to the greater accumulation of uremic toxins, acids and electrolytes, especially K^+^, in this time interval [123–125]. Based on this SLR, an increased risk of all-cause mortality was observed on the day after LIDI when compared with the day after the two short intervals. Similarly, a rise in CV mortality was evident on the day after LIDI when compared with the day after SIDI or any other day of the week. Some – but not all – studies also reported an elevated mortality on the last day of LIDI (usually 60–72 h after the last HD session). The findings from this SLR indicate that LIDI is a time of increased risk among many patients receiving HD and highlights the need for further research into dialysis patterns (twice vs thrice weekly), to determine the frequency of HD with the lowest risk of adverse outcomes.

This review was limited by the high heterogeneity of studies identified which may affect the generalizability of the findings. Included studies were heterogeneous regarding the study population and cohort size (5–74,219 patients) by selecting specific patient populations alongside CKD (including HCV or HIV-infected, the presence of pruritus, obese, with hyperparathyroidism or leprosy) and by estimating the frequency of HK associated with medications administered to treat comorbidities or interventions to control K^+^ levels. In addition, the S-K^+^ threshold used to define HK varied significantly between studies (ranging from 5.0–6.0 mmol/L with many studies not providing a definition) making difficult to stratify outcomes based on HK severity. Furthermore, some of the studies included in the analysis reported HK as an adverse event of interventions assessed in clinical trials (this may underestimate its frequency and it is difficult to interpret due to the lack of information concerning the definition of HK). Information regarding follow-up, HD vintage and weekly schedule were sparse throughout the studies. Specifically, for studies reporting on mortality, in addition to population heterogeneity, the wide range of outcome measures used and the limited reference to the timepoint those outcomes were assessed at precluded pooling of data and meta-analysis was not considered appropriate.

In conclusion, evidence identified in this systematic review indicated a relationship between HK and LIDI with mortality in patients receiving HD for renal disease or acute renal failure. The majority of the evidence of this association is from observational studies of a retrospective nature, therefore further research that includes prospective studies and RCTs is required to determine if HK plays a causal role in the observed associations. In the meantime, improved monitoring and management of S-K^+^ levels in patients with renal disease may have the potential to improve outcomes in a chronic and an emergency HD setting.

## Supplementary Material

Supplemental Material
