# Supplementary material for: The effect of hyperkalemia and long inter-dialytic interval on morbidity and mortality in patients receiving hemodialysis: a systematic review
Source: Ren Fail. 2021 Jan 21;43(1):241–54. doi: 10.1080/0886022X.2020.1871012 (PMC7833048; doi:10.1080/0886022X.2020.1871012)
Supplement: Supplemental Material [file IRNF_A_1871012_SM1290.pdf]

The effect of hyperkalaemia and long inter-dialytic interval on morbidity and mortality in patients receiving haemodialysis: a systematic review

Danai Bem, Daniel Sugrue, Ben Wilding, Ina Zile, Karin Butler, David Booth, Eskinder Tafesse, Phil McEwan

SUPPLEMENTARY MATERIAL

Table S1. Search strategy in PubMed (MEDLINE and MEDLINE In-Process) for the identification of studies reporting the impact of HK and LIDI on patients receiving HD

#	Search terms
**Intervention**	
1	hemodialysis[MeSH Terms]
2	hemodialysis[Title/Abstract] OR haemodialysis[Title/Abstract] OR hemofiltration[Title/Abstract] OR haemofiltration[Title/Abstract] OR dialysis[Title/Abstract] OR dialyses[Title/Abstract]
3	#1 OR #2
**Population**	
4	hyperkalemia[MeSH Terms]
5	hyperkalemia[Title/Abstract] OR hyperkalaemia[Title/Abstract]
6	"High potassium"[Title]
7	"inter-dialytic interval*"[Title/Abstract] OR "interdialytic interval*"[Title/Abstract] OR "intradialytic interval*"[Title/Abstract] OR LIDI[Title/Abstract]
8	#4 OR #5 OR #6 OR #7
**Combine**	
9	#3 AND #8
10	#9 AND English[Language]

Table S2. Summary of baseline characteristics and main outcomes of identified studies (n=70) reporting frequency of HK in HD patients

Author, year, country (trial ID)	Study design	HD & population specifics	Co-morbid conditions	S-K$^+$ levels (mmol/L)	Study subgroups (N)	Pre-dialysis HK		
						Timepoint	Outcome measure	Value
**ESRD population (n = 40)**								
Balamuthusamy et al. 2017, USA (Datalink)	Retrospective, observational, multicentre	Outpatients	NR	≥5.5	Total population (1022)	NR	Prevalence - Proportion of patients with HK	9.0%
				5.5–5.9	Moderate HK (346)			6.75%
				≥6.0	Severe HK (346)			2.25%
Block et al. 2016, USA (NCT01764854)	Phase 2 RCT, multicentre	Maintenance HD	NR	NR	PBO (35)	1 month	Proportion of patients with HK	0.0%
				NR	Tenapanor (37)			8.1%
Borgia et al. 2019, Canada, UK, Spain, Israel, New Zealand, Australia (NCT0306852)	Phase 2 single-arm, multicentre	Chronic HD (HCV infected)	Diabetes 32.0%; Hypertension 90.0%	NR	Sofosbuvir/velpatasvir (59)	3 months	Proportion of patients with HK (reported as drug AE)	5%
Chan et al. 2011, USA	Retrospective, observational, multicentre	Chronic HD	Diabetes 50.9%; MI or CAD 18.1%; CHF 25.1%; Cerebrovascular accident 5.0%; PVD 17.4%	≥6.5	ARB (5,828)	24 months	Proportion of patients with HK	28.1%
			Diabetes 53.2%; MI or CAD 20.0%; CHF 27.7%; Cerebrovascular accident 6.2%; PVD 18.4%	≥6.5	ACEI (22,800)		Proportion of patients with HK	30.2%
Charytan et al. 2017, USA (Spin-D; NCT02285920)	Phase 2 RCT, multicentre	Maintenance HD	NR	>6.0	PBO (51)	8 months	Incidence rate	3.5 events per 100 patient-weeks
				>6.0	SPL 12.5 mg/day (27)		Incidence rate	2.12 events per 100 patient-weeks
				>6.0	SPL 25.0 mg/day (26)		Incidence rate	2.18 events per 100 patient-weeks
				>6.0	SPL 50.0 mg/day (25)		Incidence rate	5.04 events per 100 patient-weeks
Charytan et al. 2019, USA (Spin-D; NCT02285920)	Phase 2 RCT, multicentre	Maintenance HD	Diabetes 47.1%; Hypertension 98.0%; Hyperlipidaemia 39.2%; CHF 19.6% Cerebrovascular accident 27.5%; PVD 11.8%; CHD/CAD 23.5%; Arrhythmia 13.7%	≥6.0	PBO (NR)	8 months	Frequency - Proportion of patients with HK	15.6%
			Diabetes 48.1%; Hypertension 88.9%; Hyperlipidaemia 33.3%; CHF 7.4% Cerebrovascular accident 14.8%; PVD 7.4%; CHD/CAD 3.7%; Arrhythmia 3.7%	≥6.0	SPL 12.5 mg/day (NR)		Frequency - Proportion of patients with HK	3.7%
			Diabetes 53.8%; Hypertension 88.5%; Hyperlipidaemia 38.5%; CHF 15.4% Cerebrovascular accident 3.8%; PVD 15.4%; CHD/CAD 30.8%; Arrhythmia 7.7%	≥6.0	SPL 25.0 mg/day (NR)		Frequency - Proportion of patients with HK	0.0%

Author, year, country (trial ID)	Study design	HD & population specifics	Co-morbid conditions	S-K$^+$ levels (mmol/L)	Study subgroups (N)	Pre-dialysis HK		
						Timepoint	Outcome measure	Value
			Diabetes 60.0%; Hypertension 92.0%; Hyperlipidaemia 40.0%; CHF 20.0% Cerebrovascular accident 12.0%; PVD 16.0%; CHD/CAD 28.0%	≥6.0	SPL 50.0 mg/day (NR)		Frequency - Proportion of patients with HK	12.0%
Chen et al. 2019, China (FGCL-4592-806)	Phase 3 RCT, multicentre	Chronic HD	Diabetes 15.5%	>5.5	Total population (304)	At baseline	Proportion of patients with HK	28%
				> 5.5 to 6.0	HK (52)			17.1%
				> 6.0 to 6.5	HK (19)			6.3%
				> 6.5	HK (14)			4.6%
Cho et al. 2008, Korea	Prospective, observational, multicentre	Maintenance HD	NR	>5.5	Total population (110)	NR	Prevalence - Proportion of patients with HK	43.5%
Cobo Sanchez et al. 2007, Spain	Prospective, observational (cross sectional), single centre	Haemofiltration	Diabetes 32.4%; Hypertension 91.0%; Hyperlipidaemia 23.4%; CHD/CAD 11.7%	≥5.5	Total population (34)	NR	Proportion of patients with HK	29.4%
El-Sharkawy et al. 2009, Egypt	Prospective, observational, multicentre	Maintenance HD	NR	NR	Total population (354)	NR	Prevalence	Pre-dialysis – 41.2% Post-dialysis – 6.5%
Eron et al. 2018, Europe[¶] and USA (NCT02600819)	Phase 3b single arm, multicentre	Chronic HD (HIV-1-infected with ESRD)	Diabetes 27.0%; Hypertension 95.0%; Hyperlipidaemia 42.0%; Other cardiac disease 47.0%	NR	Total population (55); elvitegravir, cobicistat, emtricitabine, and tenofovir alafenamide	22 months	Proportion of patients with HK (reported as drug AE)	15.0%
Farese et al. 2009, Switzerland (NCT00384384)	Phase 2 RCT, single centre	Chronic HD	NR	>6.0	PBO (10)	6 months	Frequency of severe HK within 358 K$^+$ measurements	9.0%
				>6.0	Glycyrrhetinic acid (10)			0.6%
Fishbane et al. 2019, USA (NCT01473420)	Phase 3 RCT, multicentre	Maintenance HD	NR	NR	Total population (244); epoetin alfa or epoetin alfa-epbx	4.6 months	Proportion of patients with HK (reported as drug AE)	1.6%
Fishbane et al. 2019b, Japan Russia, USA, UK (DIALIZE, NCT03303521)	Phase 3b RCT, multicentre	Maintenance HD	NR	>5.4	PBO (99)	2 weeks	Proportion of patients with HK (reported as drug AE)	6.1%
				>5.4	SZC (97)			2.1%
Fishbane et al. 2020, USA (KALM-1)	Phase 3 RCT, multicentre	Chronic HD (moderate-to-severe pruritus)	NR	NR	PBO (188)	3 months	Proportion of patients with HK (reported as drug AE)	2.1%
				NR	Difelikefalin (189)			2.1%

Author, year, country (trial ID)	Study design	HD & population specifics	Co-morbid conditions	S-K$^+$ levels (mmol/L)	Study subgroups (N)	Pre-dialysis HK		
						Timepoint	Outcome measure	Value
Hammer et al. 2019, Germany (MiREnDa, NCT01691053)	Phase 2 RCT, multicentre	Maintenance HD	Diabetes 32.0%; Hypertension 78.0%; PVD 20.0%; CHD/CAD 38.0%; Other cardiac disease 64.0%; Arrhythmia 10.0%	≥6.0	SPL (50)	9 months	Proportion of patients with HK	90.0%
			Diabetes 34.0%; Hypertension 97.9%; PVD 21.3%; CHD/CAD 36.2%; Other cardiac disease 48.8%; Arrhythmia 14.9%	≥6.0	PBO (47)			83.0%
Han et al. 2007, Korea	Prospective, non-RCT cross-over, multicentre	Maintenance HD	NR	≥5.6	No drug (93)	3 months	Proportion of patients with HK	77.4%
				5.6-6.0	No drug (93)			51.6%
				>6.0	No drug (93)			25.8%
				≥5.6	ACEI (47)			83.0%
				5.6-6.0	ACEI (47)			53.1%
				>6.0	ACEI (47)			29.8%
				≥5.6	ARB (46)			65.2%
				5.6-6.0	ARB (46)			45.7%
				>6.0	ARB (46)			19.6%
				≥5.6	ACEI or ARB (93)			74.2%
				5.6-6.0	ACEI or ARB (93)			49.5%
				>6.0	ACEI or ARB (93)			24.7%
				≥5.6	ACEI and ARB (62)			56.5%
				5.6-6.0	ACEI and ARB (62)			38.7%
				≥6.0	ACEI and ARB (62)			17.7%
He et al. 2018, China	Prospective, observational, multicentre	Chronic HD (acute HCV-infected)	NR	NR	Total population (33)	3 months	Proportion of patients with HK	30.3%
Hooker et al. 2015, USA	Retrospective, observational, multicentre	NR	NR	>5.3	Total population (55)	NR	Proportion of patients with HK	62.0%
				≥6.0	Severe HK (NR)			55.0%
Huang et al. 2015, Taiwan	Retrospective, observational (cohort)	Maintenance HD	Diabetes 33.7%; CHD/CAD 12.5% Arrhythmia 5.1%	>5.0	Total population (312)	NR	Proportion of patients with HK	33.3%
Hussain et al. 2003, USA	Prospective, observational, single centre	Chronic HD	Arrhythmia 6.1%	≥5.5	Total population + SPL (312)	1 month	Proportion of patients with HK	35.7%
				5.5-6.0	Moderate HK + SPL (15)			30.8%
				>6.0	Severe HK + SPL (15)			7.1%
Hwang et al. 2011, Taiwan	Retrospective, observational, single centre	Chronic HD	Diabetes 41.0%; CHF 8.0%; Cerebrovascular accident 8.0%; PVD 12.0%; CHD/CAD 20.0%	>5.5	Total population (423)	54 months	Proportion of patients with HK	12.1%
Jebali et al. 2020, Tunisia	Observational (cross-	Maintenance HD	Diabetes 24.4%; Hypertension 80.3%	≥5.5	Total population (66)	NR	Proportion of patients with HK	28.8

Author, year, country (trial ID)	Study design	HD & population specifics	Co-morbid conditions	S-K$^+$ levels (mmol/L)	Study subgroups (N)	Pre-dialysis HK		
						Timepoint	Outcome measure	Value
	sectional), multicentre							
Karaboyas et al. 2018, International** (DOPPS phase 2-5)	Prospective, observational (cohort), multicentre	Maintenance HD	Diabetes 57.0%; Hypertension 85.0%; CHF 38.0%; Cerebrovascular disease 14.0%; PVD 25.0%; CHD/CAD 45.0%; Other cardiac disease 28.0%	>5.5	N. America, short vintage no RAASI (2,845)	19.2 months*	Proportion of patients with HK	6.0%
			Diabetes 66.0%; Hypertension 92.0%; CHF 37.0%; Cerebrovascular disease 15.0%; PVD 25.0%; CHD/CAD 47.0%; Other cardiac disease 23.0%	>5.5	N. America, short vintage RAASI (1538)			7.0%
			Diabetes 35.0%; Hypertension 82.0%; CHF 26.0%; Cerebrovascular disease 14.0%; PVD 25.0%; CHD/CAD 38.0%; Other cardiac disease 30.0%	>5.5	Europe and A/NZ, short vintage no RAASI (3,288)			14.0%
			Diabetes 44.0%; Hypertension 91.0%; CHF 25%; Cerebrovascular disease 16.0%; PVD 28.0%; CHD/CAD 41.0%; Other cardiac disease 29.0%	>5.5	Europe and A/NZ, short vintage RAASI (2,245)			17.0%
			Diabetes 44.0%; Hypertension 80.0%; CHF 31.0%; Cerebrovascular disease 15.0%; PVD 13.0%; CHD/CAD 27.0%; Other cardiac disease 24.0%	>5.5	Japan, short vintage no RAASI (713)			7.0%
			Diabetes 57.0%; Hypertension 89.0%; CHF 32.0%; Cerebrovascular disease 11.0%; PVD 11.0%; CHD/CAD 25.0%; Other cardiac disease 20.0%	>5.5	Japan, short vintage RAASI (792)			10.0%
			Diabetes 54.0%; Hypertension 86.0%; CHF 39.0%; Cerebrovascular disease 17.0%; PVD 27.0%; CHD/CAD 46.0%; Other cardiac disease 30.0%	>5.5	N. America, long vintage no RAASI (7,566)			12.0%
			Diabetes 61.0%; Hypertension 92.0%; CHF 40.0%; Cerebrovascular disease 16.0%; PVD 28.0%; CHD/CAD 50.0%; Other cardiac disease 28.0%	>5.5	N. America, long vintage RAASI (5,451)			15.0%
			Diabetes 30.0%; Hypertension 78.0%; CHF 26.0%; Cerebrovascular disease 17.0%; PVD 30.0%; CHD/CAD 41.0%; Other cardiac disease 37.0%	>5.5	Europe and A/NZ, long vintage no RAASI (11,118)	19.2 months*	Proportion of patients with HK	26.0%
			Diabetes 39.0%; Hypertension 91.0%; CHF 31.0%; Cerebrovascular disease 19.0%; PVD 33.0%; CHD/CAD 46.0%; Other cardiac disease 36.0%	>5.5	Europe and A/NZ, long vintage RAASI (5,879)			30.0%
			Diabetes 29.0%; Hypertension 64.0%; CHF 19.0%; Cerebrovascular disease	>5.5	Japan, long vintage no RAASI (3,986)			19.0%

Author, year, country (trial ID)	Study design	HD & population specifics	Co-morbid conditions	S-K+ levels (mmol/L)	Study subgroups (N)	Pre-dialysis HK		
						Timepoint	Outcome measure	Value
			14.0%; PVD 16.0%; CHD/CAD 32.0%; Other cardiac disease 32.0%					
			Diabetes 42.0%; Hypertension 88.0%; CHF 20.0%; Cerebrovascular disease 14.0%; PVD 16.0%; CHD/CAD 32.0%; Other cardiac disease 27.0%	>5.5	Japan, long vintage RAASI (3,124)			24.0%
Karaboyas et al. 2017, International‡ (DOPPS all phases)	Prospective, observational (cohort), multicentre	Maintenance HD	Diabetes 43.0%; Hypertension 84.0%; CHF 33.0%; Cerebrovascular disease 15.0%; PVD 26.0%; CHD/CAD 42.0%; Other cardiac disease 31.0%	>5.0	Total population (55,183)	16.5 (8.1–25.5) months†	Proportion of patients with HK	38.0%
				5.1-5.5	Mild HK (10,700)			19.0%
				5.6-6.0	Moderate HK (6,259)			11.0%
				>6.0	Severe HK (4,399)			8.0%
Knoll et al. 2002, Canada	Prospective, observational (cohort), single centre	Chronic HD	Diabetes 24.0%	≥5.5	ACEI (62)	8 months*	Incidence rate	17.0 per 100 person-months
			Diabetes 19.0%	≥5.5	Controls (158)			26.0 per 100 person-months
Kourtellidou et al. 2016, UK (BicHD; NCT02692378)	Phase 4 RCT	In-centre HD	NR	>6.0	Without sodium bicarbonate (16)	At baseline	Proportion of patients with HK	12.1%
				>6.0	With sodium bicarbonate (16)	3 months		4.9%
Lee et al. 2015, Korea	Retrospective, observational, single centre	Maintenance HD	Hypertension 81.9%; CHF 19.3%	NR	Total population (409)	NR	Proportion of patients with HK	3.9%
Megahed et al. 2019, Saudi Arabia	Observational, single centre	Maintenance HD	NR	NR	Total population (93)	NR	Proportion of patients with HK	12.9%
Movilli et al. 2018, Italy	Retrospective, observational, single centre	Chronic HD (oligo-anuric patients)	Diabetes 18.0%; Other cardiac disease 40.0%	>6.0	No ACEI/ARB - mild HK (112)	At baseline	Proportion of patients with mild and severe HK before and after ACEi/ARB	18%
					No ACEI/ARB - severe HK (112)			0.0%
				5.5-6.0	With ACEI/ARB - mild HK (112)	3 months		52.0%
				>6.0	With ACEI/ARB - severe HK (112)			19.0%
Nemati et al. 2010, Iran	Observational (cross-sectional), multicentre	Maintenance HD	Arrhythmia 0.0%	>5.2	Total population (80)	NR	Prevalence - Proportion of patients with HK	59.0%
Raza et al. 2004, Saudi Arabia	Prospective, single centre	Maintenance HD	NR	>6.2	Before nutritional counselling (110)	NR	Proportion of patients with HK	21.0%
				>6.2	After nutritional counselling (110)			9.0%

Author, year, country (trial ID)	Study design	HD & population specifics	Co-morbid conditions	S-K+ levels (mmol/L)	Study subgroups (N)	Pre-dialysis HK		
						Timepoint	Outcome measure	Value
Singh et al. 2017, USA	Prospective, observational	NR	NR	>5.0	Dialysate K+ <3.0 mEq/L (179)	12 days	Proportion of patients with HK after LIDP	50.0%
				>5.0	Dialysate K+ ≥3.0 mEq/L (33)			36.0%
Thomas et al. 2020, Canada	Retrospective, observational (cohort), multicentre	Maintenance HD	Diabetes: 60.9%; Hypertension: 89%; MI: 14.2%; CHF 44.4%; Cerebrovascular accident 6.5%; PVD 17.5%; CHD/CAD: 61.3%; Arrhythmia 20.5%	NR	Prevalence cohort-monthly testing (5,335)	35.8 (14-17'5) †	Prevalence - Proportion of patients with HK	Monthly testing: 7%<br>Every 6 weeks: 6%
							Incidence rate	Monthly testing: 22.6<br>Every 6 weeks: 17.6<br>per 1000 patient years
			Diabetes: 63.3%; Hypertension: 83.7%; MI: 11.2%; CHF 43.2%; Cerebrovascular accident 4.9%; PVD 6.9%; CHD/CAD: 51.4%; Arrhythmia 17.3%	NR	Incidence cohort-monthly testing (7,752)	21.1 (7.6-37.2) †	Prevalence - Proportion of patients with HK	Monthly testing: 4%<br>Every 6 weeks: 3%
							Incidence rate	Monthly testing: 21.0<br>Every 6 weeks: 14.3<br>per 1000 patient years
Tzamaloukas et al. 1987, USA	Retrospective, observational, single centre	Chronic HD	NR	>6.0	Non-diabetic patients (136)	50.4 (54.0) months*	Proportion of patients with HK	10.0%
				>6.0	Diabetic patients (36)	26.4 (20.4) months *		8.7%
Vaz de Melo Ribeiro et al. 2020, Brazil	Prospective, single centre	NR	NR	NR	Prior to nutritional intervention	At baseline	Proportion of patients with HK	56.6%
					After the nutritional intervention	4 months		28.9%
Vukusich et al. 2010, Chile	RCT	Chronic HD	Cerebrovascular accident 8.7%	NR	PBO (23)	24 months*	Proportion of patients with HK	0.0%
			Cerebrovascular accident 10.0%	NR	SPL (30)			0.0%
Walsh et al. 2015, Canada (PHASE)	RCT, multicentre	NR	Diabetes 51.9%; CHF 10.4%; Cerebrovascular accident: 5.2%; PVD 6.5%; CHD/CAD 20.8%	>6.0	Eplerenone (75)	3 months	Proportion of patients with HK	24.7%
			Diabetes 51.9%; CHF 7.8%; Cerebrovascular accident: 5.2%; PVD 11.7%; CHD/CAD 16.9%	>6.0	PBO (71)			18.2%
			NR	>6.5	Eplerenone (NR)			11.7%
				>6.5	PBO (NR)			2.6%
Woods et al. 2019, Australia	Prospective	HD patients with BMIs >30	Diabetes 45.5%	>6.0	Total population (22)	3 months	Proportion of patients with HK	54.5%
Yusuf et al. 2016, USA (USRDS)	Retrospective, observational (cohort), multicentre	Maintenance HD	Diabetes: 57.9%; Atherosclerotic heart disease 31.0%; CHF 38.1%; Cerebrovascular accident 12.2%; PVD 21.9%; Other cardiac disease 11.6%; Arrhythmia 14.9%	≥5.5	2007 cohort (28,774)	9 months* (135,354 months§§)	Incidence rate	16.7 per 100 person-months

Author, year, country (trial ID)	Study design	HD & population specifics	Co-morbid conditions	S-K+ levels (mmol/L)	Study subgroups (N)	Pre-dialysis HK		
						Timepoint	Outcome measure	Value
			Diabetes: 59.0%; Atherosclerotic heart disease 31.3%; CHF 39.1%; Cerebrovascular accident 11.9%; PVD 22.1%; Other cardiac disease 13.1%; Arrhythmia 15.0%	≥5.5	2008 cohort (34,788)			16.8 per 100 person-months
			Diabetes: 59.9%; Atherosclerotic heart disease 31.5%; CHF 38.7%; Cerebrovascular accident 12.1%; PVD 22.4%; Other cardiac disease 14.3%; Arrhythmia 14.7%	≥5.5	2009 cohort (34,571)			16.5 per 100 person-months
			Diabetes: 60.3%; Atherosclerotic heart disease 30.4%; CHF 38.0%; Cerebrovascular accident 12.3%; PVD 22.1%; Other cardiac disease 14.6%; Arrhythmia 14.6%	≥5.5	2010 cohort (36,888)			16.3 per 100 person-months
**CKD population (n = 21)**								
Acha et al. 2020, Cameroon	Prospective, observational (cross-sectional), single centre	Maintenance HD	Diabetes 14.7%	≥5.5	Total population (95)	4 months	Prevalence - Proportion of patients with HK	33.7%
Belmar et al. 2019, Spain	Retrospective, observational, single centre	NR	Diabetes 31.7%; CHF 5.0%; Cerebrovascular accident 5.0%; CHD/CAD 13.9%	>5.5	Total population (1097)	3.2 months*	Prevalence - Proportion of patients with HK	16.4%
				5.5-5.9	Mild HK			8.7%
				6.0-6.4	Moderate HK			4.4%
				6.5-6.9	Severe			2.0%
				≥7.0	Very severe			1.4%
Block et al. 2019, International[§] (NCT01785849, NCT01788046)	Phase 3 RCTs, multicentre	With secondary hyperparathyroidism	NR	NR	PBO – all HK (503)	6 months	Proportion of patients with HK (reported as drug AE)	2.1%
				NR	Etelcalcetide – all HK (513)			4.4%
				NR	PBO – severe HK			0.4%
				NR	Etelcalcetide – severe HK			2%
Checherita et al. 2011, Romania	Non-RCT, single centre	NR	NR	>5.0	Total population (202)	24 months	Proportion of patients with HK	57.9%
de Almeida et al. 2018, Brazil	Observational (cross-sectional), single centre	NR	NR	>6.0	With residual renal function (27)	NR	Proportion of patients with HK	36.8%
				>6.0	Without residual renal function (24)			82.7%
Frazão et al. 2014, Brazil	Observational (cross-sectional), single centre	NR	NR	NR	Total population (330)	NR	Proportion of patients with HK	64.6%

Author, year, country (trial ID)	Study design	HD & population specifics	Co-morbid conditions	S-K$^+$ levels (mmol/L)	Study subgroups (N)	Pre-dialysis HK		
						Timepoint	Outcome measure	Value
Garagarza et al. 2015, Portugal	Observational (longitudinal cohort), multicentre	Maintenance HD	Diabetes 34.4%	>5.5	Without nutritional counselling (660)	At baseline	Proportion of patients with HK	52.0%
				>5.5	With nutritional counselling (660)	6 months		35.8%
Genovesi et al. 2009, Italy	Retrospective, observational, multicentre	Chronic HD	Diabetes 19.1%; Hypertension 81.3%; CHD/CAD 23.5%; Arrhythmia 26.7%	≥6.0	Total population (476)	36 months	Proportion of patients with HK	31.3%
Iseki et al. 2013, Japan (OCTOPUS; CRG010600030)	RCT, multicentre	Chronic HD	Diabetes 32.0%; MI 3.0%; Cerebrovascular accident 15.0%	NR	Control (234)	42 months	Proportion of patients with HK (reported as drug AE)	0.9%
			Diabetes 32.0%; MI 6.0%; Cerebrovascular accident 15.0%	NR	ARB Olmesartan (235)			0.9%
Kim et al. 2017, USA (DaVita)	Observational (cohort), multicentre	NR	Diabetes 58.0%; Hypertension 61.0%; Atherosclerotic heart disease 13.0%; CHF 36.0%; Other cardiac disease 13.0%	5.0-5.5	Af-Am patients (2,384)	60 months	Proportion of patients with HK	6.9%
				>5.0	Af-Am patients (34,574)			5.5%
				>5.5	Af-Am patients (509)			1.5%
			Diabetes 55.0%; Hypertension 48.0%; Atherosclerotic heart disease 17.0%; CHF 37.0%; Other cardiac disease 18.0%	5.0-5.5	White patients (5,228)			10.2%
				>5.0	White patients (51,297)			12.5%
				>5.5	White patients (1204)			2.3%
			Diabetes 68.0%; Hypertension 45.0%; Atherosclerotic heart disease 12.0%; CHF 38.0%; Other cardiac disease 12.0%	5.0-5.5	Hispanic patients (2,572)			15.7%
				>5.0	Hispanic patients (16,370)			21.2%
				>5.5	Hispanic patients (902)			5.5%
Kovesdy et al. 2007, USA (DaVita)	Observational (cohort), multicentre	Maintenance HD	NR	≥5.0	Total population (74,219)	NR	Proportion of patients with HK	39.5%
				5.0-5.9	Mild HK			35%
				>6.0	Severe HK			4.5%
Kovesdy et al. 2019b, USA (DaVita)	Retrospective, observational (cohort), multicentre	Chronic HD	Diabetes 69.0%; MI 8.0%; CHF 15.0%; PVD 10.0%; Arrhythmia 2.0%	≥6.0	Pre - PAT (527)	4.6 months†	Proportion of patients with HK	48%
				≥6.0	Post - PAT (392)			22%
Lin et al. 2009, China	Observational (cross-sectional), single centre	Chronic HD	Diabetes 41.5%	≥5.3	Total population (200)	NR	Proportion of patients with HK	35.0%
Mattos et al. 2012, Brazil	NR	NR	Diabetes 50.0%; Hypertension 100.0%	NR	Total population (49)	6 months	Proportion of patients with HK	43.0%

Author, year, country (trial ID)	Study design	HD & population specifics	Co-morbid conditions	S-K$^+$ levels (mmol/L)	Study subgroups (N)	Pre-dialysis HK		
						Timepoint	Outcome measure	Value
Patil et al. 2015, India	Retrospective, observational, single centre	CKD stages 2-5 requiring HD	Diabetes 27.5%; Hypertension 45.5%	NR	Total population (51)	NR	Proportion of patients with HK	25.5%
Poulikakos et al. 2012, UK	Prospective	Maintenance HD	NR	>6.0	Controls (77)	NR	Proportion of patients with HK	5.0%
				>6.0	With Beta-blockers (77)			6.0%
Pourfarziani et al. 2008, Iran	Observational (cross-sectional), multicentre	NR	NR	NR	Total population (338)	NR	Proportion of patients with HK	58.0%
Robertson et al. 2009, USA (DaVita)	Observational (cohort), multicentre	NR	NR	>5.5	Total population (NR)	12 months	Proportion of patients with HK >3 months	23.0%
Ross et al. 2015, USA	Prospective, observational, single centre	Outpatient	Diabetes 85.0%	≥5.7	Total population (167)	NR	Proportion of patients with HK	14.4%
				5.7-6.3	Moderate HK (167)			9.5%
				>6.3	Severe HK (167)			4.8%
Taheri et al. 2009, Iran (RALES)	RCT, single centre	Chronic HD (with heart failure)	Diabetes 62.5%; Hypertension 75.0%; Hyperlipidaemia 30.5%; CHD/CAD 62.5%	>5.5	SPL (5)	6 months	Proportion of patients with HK	0.0%
			Diabetes 62.5%; Hypertension 100.0%; Hyperlipidaemia 25.0%; CHD/CAD 50.0%	>5.5	PBO (6)			16.7%
Yang et al. 2019, China	Retrospective, observational, single centre	Maintenance HD	NR	≥5.5	Total population (204)	NR	Proportion of patients with HK	36.3%
**AKI population (n = 4)**								
Brown et al. 2019, South Africa	Retrospective, observational, single centre	HD in ICU	NR	NR	Total population (76)	NR	Patients with HK as indication for initiation of HD	5.3%
Douvris et al. 2018, Canada	Retrospective, observational, single centre	Intermittent HD while hospitalized	Diabetes 59.0%; Hypertension 68.0%; CHF 41.0%; PVD 16.0%	≥5.5	Total population (80)	12-month period	Patients with HK as indication for initiation of HD	35.0%
				≥6.0				
Maoujoud et al. 2014, Morocco	Retrospective, observational, single centre	Intermittent HD in ICU	Diabetes 44.0%; Hypertension 33.4%	7.3 (0.7)*	Total population (105)	60-month period	Patients with HK as indication for initiation of HD	48.5%
Nyandwi et al. 2020, Burundi	Retrospective, observational, multicentre	HD in ICU	NR	severe	Total population (46)	NR	Patients with HK as indication for initiation of HD	19.6%
**HK as a medical emergency (n = 5)**								
Ahmad et al. 2010, Saudi Arabia	Prospective, observational, single centre	Emergency HD	NR	>5.5	Total population (192)	At baseline	Proportion of patients with HK	11.5%

Author, year, country (trial ID)	Study design	HD & population specifics	Co-morbid conditions	S-K$^+$ levels (mmol/L)	Study subgroups (N)	Pre-dialysis HK		
						Timepoint	Outcome measure	Value
Rafique et al. 2020, UK	Observational, single centre	Emergency HD	NR	≥5.5	Total population (66)	NR	Proportion of patients with HK	59%
				5.5-6.4	Moderate HK (19)			29%
				>6.5	Severe HK (20)			30%
Sacchetti et al. 1999, USA	Retrospective, observational, single centre	Emergency HD	CHF 65.0%	NR	Total population (37)	NR	Proportion of patients with HK	24.0%
Triqui et al. 2020, Tunisia	Observational (cross-sectional), single centre	Emergency HD	Diabetes 45.0%; Hypertension 58.0%	NR	Total population (117)	NR	Patients with HK as indication for initiation of HD	37.6
Yalin et al. 2012, Turkey	Retrospective, observational	Emergency HD	Diabetes 24.7%; Hypertension 36.2%	NR	Surviving patients (489)	NR	Proportion of patients with HK	21.1%

A/NZ: Australia/New Zealand; ACEI: angiotensin-converting-enzyme inhibitor; Af-Am: African-American; AKI: acute kidney injury; ARB: angiotensin II receptor blockers; CAD: coronary artery disease; CHD: coronary heart disease; CHF: congestive heart failure; CKD: chronic kidney disease; ESRD: end-stage renal disease; HCV: hepatitis C virus; HD: haemodialysis; HIV-1: human immunodeficiency virus 1; HK: hyperkalaemia; ICU: intensive care unit; LIPD: long interdialytic period; MI: myocardial infarction; NR: not reported; PAT: patiromer; PBO: placebo; PVD: peripheral vascular disease; RAASI: renin-angiotensin-aldosterone system inhibitors; RCT: randomised controlled trial; S-K$^+$: serum potassium; SPL: spironolactone; SPS: sodium polystyrene sulphonate

* mean (SD)

† median (IQR)

§ Australia, Austria, Belgium, Canada, the Czech Republic, Denmark, Estonia, France, Germany, Greece, Hungary, Israel, Italy, Latvia, Lithuania, New Zealand, Poland, Portugal, the Russian Federation, Spain, Sweden, Switzerland, Turkey and USA

¶ Austria, France, Germany and USA

** North America (Canada, USA), Europe/ANZ (Belgium, France, Germany, Italy, Spain, Sweden, United Kingdom, Australia, New Zealand) and Japan

‡ France, Germany, Italy, Japan, Spain, United Kingdom, & USA in phases 1 to 5; Australia and New Zealand, Belgium, Canada, and Sweden in phases 2 to 5; and the Gulf Cooperation Council (including Bahrain, Kuwait, Oman, Qatar, Saudi Arabia and United Arab Emirates), China, Russia, and Turkey in phase 5

§§ cumulative follow-up time

Table S3. Summary of baseline characteristics and main outcomes of identified studies (n=25) reporting mortality outcomes associated with HK

Author, year, country (trial ID)	Study design	Population	Co-morbid conditions	S-K$^+$ levels (mmol/L)	Subgroup population (N)	Deaths due to HK (%)	All-cause mortality		CV- mortality		CV events
							Timepoint	Value	Timepoint	Value	
Alagusundaramoorthy et al. 2018, USA	Retrospective, observational, single centre	ESRD on HD	NR	7.0	Severe HK (346)	NR	NR	6.9%	NR	NR	NR
Balle et al. 1990, Germany	Retrospective, observational, single centre	End-stage renal failure due to analgesic-associated nephropathy on regular HD	Hypertension 44.0%; MI 4.0%; Hyperlipidaemia 62%; CHF 9.0%; CHD/CAD 29.0%; Other cardiac disease 62.0%; Arrhythmia 9.0%	NR	Total population (55)	7.3	NR	NR	NR	NR	NR
Chaaban et al. 2013, United Arab Emirates	Retrospective, observational (case-control)	CKD on HD	Diabetes 46.0%	NR	Calcium resonium – A (28)	0	NR	NR	NR	NR	NR
			Diabetes 57.5%	NR	Calcium resonium – NA (42)	0	NR	NR	NR	NR	NR
			Diabetes 47.0%	NR	Controls (30)	0	NR	NR	NR	NR	NR
Chan et al. 2011, USA	Retrospective, observational, multicentre	ESRD on chronic HD	Diabetes 50.9%; MI or CAD 18.1%; CHF 25.1%; Cerebrovascular accident 5.0%; PVD 17.4%	≥6.5	ARB (5,828)	NR	2 years	HR (95% CI): 0.97 (0.92-1.01)	NR	NR	NR
			Diabetes 53.2%; MI or CAD 20.0%; CHF 27.7%; Cerebrovascular accident 6.2%; PVD 18.4%	≥6.5	ACEI (22,800)	NR	2 years	HR (95% CI): 1.0	NR	NR	NR
Charytan et al. 2017, USA (Spin-D; NCT02285920)	Phase 2 RCT, multicentre	ESRD on maintenance HD	NR	>6.0	PBO (51)	0	NR	NR	NR	NR	NR
				>6.0	SPL 12.5 mg/day (27)	0	NR	NR	NR	NR	NR
				>6.0	SPL 25.0 mg/day (26)	0	NR	NR	NR	NR	NR
				>6.0	SPL 50.0 mg/day (25)	0	NR	NR	NR	NR	NR

Author, year, country (trial ID)	Study design	Population	Co-morbid conditions	S-K^+ levels (mmol/L)	Subgroup population (N)	Deaths due to HK (%)	All-cause mortality		CV- mortality		CV events
							Timepoint	Value	Timepoint	Value	
Chatoth et al. 2018, USA	Retrospective, observational, multicentre	ESRD on HD	NR	>6.0	No K^+ binder (NR)	NR	6 months	2.5%	NR	NR	NR
				>5.5	No K^+ binder (26400)	NR	6 months	2.3%	NR	NR	NR
				>6.0	PAT (NR)	NR	6 months	0.0%	NR	NR	NR
				>5.5	PAT (106)	NR	6 months	2.8%	NR	NR	NR
				>6.0	SPS (NR)	NR	6 months	1.8%	NR	NR	NR
				>5.5	SPS (649)	NR	6 months	2.5%	NR	NR	NR
Ferrey et al. 2018, USA<br>(MADRAD, NCT01415570)	Prospective, observational (cohort), multicentre	CKD on HD	Diabetes 50.0%; CHF 13.0%; Cerebrovascular accident 2.0%; CHD/CAD 13.0%; CVD 23.0%	≥5.0	Dialysate K^+ 1.0 mEq/L (47)	NR	3.2 years[¶]	HR (95% CI): 2.74 (1.39-5.40)	NR	NR	NR
				<5.0	Dialysate K^+ 1.0 mEq/L (47)	NR	3.2 years[¶]	HR (95% CI): 0.76 (0.27-2.14)	NR	NR	NR
			Diabetes 52.0%; CHF 10.0%; Cerebrovascular accident 0.0%; CHD/CAD 9.0%; CVD 18.0%	≥5.0	Dialysate K^+ 2.0 mEq/L (449)	NR	3.2 years[¶]	HR (95% CI): 1.0	NR	NR	NR
				<5.0	Dialysate K^+ 2.0 mEq/L (449)	NR	3.2 years[¶]	HR (95% CI): 1.0	NR	NR	NR
			Diabetes 50.0%; CHF 9.0%; Cerebrovascular accident 1.0%; CHD/CAD 13.0%; CVD 19.0%	≥5.0	Dialysate K^+ 3.0 mEq/L (128)	NR	3.2 years[¶]	HR (95% CI): 0.75 (0.33-1.69)	NR	NR	NR
				<5.0	Dialysate K^+ 3.0 mEq/L (128)	NR	3.2 years[¶]	HR (95% CI): 0.95 (0.59-1.52)	NR	NR	NR
Genovesi et al. 2009, Italy	Retrospective, observational, multicentre	CKD on chronic HD	Diabetes 19.1%; Hypertension 81.3%; CHD/CAD 23.5%; Arrhythmia 26.7%	≥6.0	With HK (149)	NR	3 years[§]	cumulative incidence: 40.7%	3 years[§]	cumulative incidence: 18.2%	NR
								HR (95% CI): 2.74 (1.28-5.85)			
				<6.0	Without HK (327)	NR	3 years[§]	cumulative incidence: 31.9%	3 years[§]	cumulative incidence: 12.6%	NR
								HR (95% CI): 1.0			

Author, year, country (trial ID)	Study design	Population	Co-morbid conditions	S-K+ levels (mmol/L)	Subgroup population (N)	Deaths due to HK (%)	All-cause mortality		CV- mortality		CV events
							Timepoint	Value	Timepoint	Value	
Hwang et al. 2011, Taiwan	Retrospective, observational, single centre	ESRD on chronic HD	Diabetes 41.0%; CHF 8.0%; Cerebrovascular accident 8.0%; PVD 12.0%; CHD/CAD 20.0%	>5.5	With HK (51)	NR	4.5 years§	21.6%	4.5 years§	9.8%	Cardiac arrest 3.9%; CAD 5.9%
			Diabetes 38.0%; CHF 2.0%; Cerebrovascular accident 11.0%; PVD 6.0%; CHD/CAD 17.0%	5.5-3.5	Without HK (326)	NR	4.5 years§	18.7%	4.5 years§	5.5%	Cardiac arrest 1.8%; CAD 1.8%
			Diabetes 52.0%; CHF 7.0%; Cerebrovascular accident 20.0%; PVD 9.0%; CHD/CAD 28.0%	<3.5	With hypokalaemia (46)	NR	4.5 years§	39.1%	4.5 years§	17.3%	Cardiac arrest 6.5%; CAD 6.5%
Jadoul et al. 2012, International* (DOPPS)	Prospective, observational (cohort), multicentre	ESRD on maintenance HD	NR	>5.0	Total population (37,765)	1.3	NR	NR	NR	NR	NR
Karaboyas et al. 2017, International† (DOPPS)	Prospective, observational (cohort), multicentre	ESRD on maintenance HD	Diabetes 43.0%; Hypertension 84.0%; CHF 33.0%; Cerebrovascular disease 15.0%; PVD 26.0%; CHD/CAD 42.0%; Other cardiac disease 31.0%	5.1-5.5	Mild HK (10,700)	NR	1.4 years**	HR (95% CI): 1.02 (0.97-1.07)	NR	NR	Cardiac arrhythmia (HR: 1.0)
				5.6-6.0	Moderate HK (6,259)	NR	1.4 years**	HR (95% CI): 1.13 (1.06-1.20)	NR	NR	Cardiac arrhythmia (HR: 1.07)
				>6.0	Severe HK (4,399)	NR	1.4 years**	HR (95% CI): 1.12 (1.04-1.21)	NR	NR	Cardiac arrhythmia (HR: 1.21)
				4.0-5.0	Without HK (27,525)	NR	1.4 years**	HR (95% CI): 1.0	NR	NR	Cardiac arrhythmia (HR: 1.0)
				<4.0	With hypokalaemia (6,300)	NR	1.4 years**	HR (95% CI): 1.03 (0.97-1.09)	NR	NR	Cardiac arrhythmia (HR: 0.94)
Kim et al. 2017, USA (DaVita)	Observational (cohort), multicentre	CKD on HD	Diabetes 58.0%; Hypertension 61.0%; Atherosclerotic	≤ 3.6	White (1957)	NR	1.3 years**	37.7%	1.3 years**	11.6%	NR
				3.6-4.0	White (8,467)	NR	1.3 years**	33.7%	1.3 years**	11.0%	NR
				4.0-4.5	White (19,767)	NR	1.3 years**	32.1%	1.3 years**	11.0%	NR

Author, year, country (trial ID)	Study design	Population	Co-morbid conditions	S-K+ levels (mmol/L)	Subgroup population (N)	Deaths due to HK (%)	All-cause mortality		CV- mortality		CV events
							Timepoint	Value	Timepoint	Value	
			heart disease 13.0%; CHF 36.0%; Other cardiac disease 13.0%	4.5-5.0	White (14,674)	NR	1.3 years**	32.3%	1.3 years**	11.2%	NR
				5.0-5.5	White (5,228)	NR	1.3 years**	32.6%	1.3 years**	11.3%	NR
				>5.5	White (1204)	NR	1.3 years**	35.4%	1.3 years**	13.0%	NR
			Diabetes 55.0%; Hypertension 48.0%; Atherosclerotic heart disease 17.0%; CHF 37.0%; Other cardiac disease 18.0%	≤ 3.6	Af-Am (2,323)	NR	1.3 years**	30.4%	1.3 years**	9.2%	NR
				3.6-4.0	Af-Am (7,642)	NR	1.3 years**	23.3%	1.3 years**	8.2%	NR
				4.0-4.5	Af-Am (13,762)	NR	1.3 years**	20.9%	1.3 years**	7.3	NR
				4.5-5.0	Af-Am (7,949)	NR	1.3 years**	19.8%	1.3 years**	7.1%	NR
				5.0-5.5	Af-Am (2,384)	NR	1.3 years**	20.6%	1.3 years**	8.1%	NR
				>5.5	Af-Am (509)	NR	1.3 years**	27.5%	1.3 years**	9.2%	NR
			Diabetes 68.0%; Hypertension 45.0%; Atherosclerotic heart disease 12.0%; CHF 38.0%; Other cardiac disease 12.0%	≤ 3.6	Hispanic (431)	NR	1.3 years**	36.7%	1.3 years**	11.4%	NR
				3.6-4.0	Hispanic (1959)	NR	1.3 years**	23.7%	1.3 years**	9.9%	NR
				4.0-4.5	Hispanic (5,412)	NR	1.3 years**	19.4%	1.3 years**	8.2%	NR
				4.5-5.0	Hispanic (5,094)	NR	1.3 years**	16.9%	1.3 years**	7.1%	NR
				5.0-5.5	Hispanic (2,572)	NR	1.3 years**	16.2%	1.3 years**	7.0%	NR
				>5.5	Hispanic (902)	NR	1.3 years**	15.0%	1.3 years**	6.1%	NR
Kovesdy et al. 2007, USA (DaVita)	Observational (cohort), multicentre	CKD On maintenance HD	NR	5.0-<5.3	With HK (11,621)	NR	3 years§	30.0%	3 years§	13.0%	NR
				5.3-<5.6	With HK (8,376)	NR	3 years§	31.0%	3 years§	13.0%	NR
				5.6-<6.0	With HK (6,082)	NR	3 years§	32.0%	3 years§	13.0%	NR
				6.0-<6.3	With HK (1900)	NR	3 years§	33.0%	3 years§	14.0%	NR
				6.3-<6.6	With HK (721)	NR	3 years§	33.0%	3 years§	13.0%	NR
				≥6.6	With HK (359)	NR	3 years§	32.0%	3 years§	14.0%	NR
				≥5.6	With HK (9,062)	NR	3 years§	33.0%	3 years§	13.0%	NR
				<4.0	With hypokalaemia (6,617)	NR	3 years§	31.0%	3 years§	11.0%	NR

Author, year, country (trial ID)	Study design	Population	Co-morbid conditions	S-K+ levels (mmol/L)	Subgroup population (N)	Deaths due to HK (%)	All-cause mortality		CV- mortality		CV events
							Timepoint	Value	Timepoint	Value	
				4.0-<4.3	Without HK (8,344)	NR	3 years[§]	28.0%	3 years[§]	10.0%	NR
				4.3-<4.6	Without HK (11,917)	NR	3 years[§]	29.0%	3 years[§]	11.0%	NR
				4.6-<5.0	Without HK (18,282)	NR	3 years[§]	28.0%	3 years[§]	11.0%	NR
Li et al. 2012, China	Retrospective, observational, single centre	ESRD on maintenance HD	NR	NR	Total population (268)	4.5	NR	NR	NR	NR	NR
Li et al. 2020, China	Retrospective, observational, single centre	CKD on maintenance HD	Diabetes 63.8%; Hypertension 33.3%; other cardiac disease 26.2%	NR	Total population (210)	4.4	NR	NR	NR	NR	NR
Lomonte et al. 2004, Italy	Retrospective, observational, single centre	ESRD/ Leprosy on HD	NR	NR	Total population (8)	12.5	NR	NR	NR	NR	NR
Morduchowicz et al. 1992, Israel	Retrospective, observational	End-stage renal failure on HD	NR	NR	Total population (84)	5	NR	NR	NR	NR	NR
Onuigbo et al. 2013, Italy	Prospective, observational	ESRD on HD	NR	NR	Total population (466)	3	NR	NR	NR	NR	NR
Poulikakos et al. 2015, UK	Pilot study	CKD on maintenance HD	Diabetes 37.0%	NR	Total population (75)	1.3	NR	NR	NR	NR	NR
Pun et al. 2012, USA (DaVita)	Retrospective, observational (cohort), multicentre	ESRD on maintenance HD	Diabetes 55.2%; CHF 46.2%; CHD/CAD 34.5%; Arrhythmia 13.7%	NR	Total population (363)	1.3	NR	NR	NR	NR	NR
Shibata et al. 1983, Japan	Retrospective, single centre	CKD on HD	Diabetic nephropathy 100%	NR	Total population (62)	3.2	NR	NR	NR	NR	NR
Trajceska et al. 2020, Macedonia	Observational (cohort), single centre	ESRD on HD	NR	≥5.5	With HK (82)	NR	3 years	19.5	NR	NR	NR
Yalin et al. 2012, Turkey	Retrospective	Renal failure on emergency HD	Diabetes 23.2%; Hypertension 32.5%	NR	With HK (177)	NR	NR	41.8%	NR	NR	NR

Author, year, country (trial ID)	Study design	Population	Co-morbid conditions	S-K^+ levels (mmol/L)	Subgroup population (N)	Deaths due to HK (%)	All-cause mortality		CV- mortality		CV events
							Timepoint	Value	Timepoint	Value	
Yusuf et al. 2016, USA (USRDS)	Retrospective, cohort, multicentre	ESRD on maintenance HD	NR	≥5.5	With HK (NR)	NR	9 months§	HR (95% CI): 0.99 (0.89-1.09)	NR	NR	NR
				≥5.6	With HK (NR)	NR	9 months§	HR (95% CI): 1.03 (0.92-1.15)	NR	NR	NR
				≥5.7	With HK (NR)	NR	9 months§	HR (95% CI): 1.13 (1.01-1.28)	NR	NR	NR
				≥5.8	With HK (NR)	NR	9 months§	HR (95% CI): 1.18 (1.03-1.35)	NR	NR	NR
				≥5.9	With HK (NR)	NR	9 months§	HR (95% CI): 1.29 (1.12-1.50)	NR	NR	NR
				≥6.0	With HK (NR)	NR	9 months§	HR (95% CI): 1.37 (1.16-1.62)	NR	NR	NR
Zulham et al. 2019, Indonesia	Observational, single centre	Diabetic ESRD on HD	Diabetes 100%	NR	With HK (96)	NR	NR	49.0	NR	NR	NR
				NR	Without HK (115)	NR	NR	25.2	NR	NR	NR

A: adherent group; A/NZ: Australia/New Zealand; ACEI: angiotensin-converting-enzyme inhibitor; Af-Am: African-American; ARB: angiotensin II receptor blockers; CAD: coronary artery disease; CHD: coronary heart disease; CHF: congestive heart failure; CI: confidence interval; CKD: chronic kidney disease; ESRD: end-stage renal disease; HD: haemodialysis; HK: hyperkalaemia; HR: hazard ratio; MI: myocardial infarction; NA: non-adherent group; NR: not reported; PAT: patiromer; PBO: placebo; PVD: peripheral vascular disease; RCT: randomised controlled trial; SPL: spironolactone; SPS: sodium polystyrene sulphonate

* Australia, Belgium, Canada, France, Germany, Italy, Japan, New Zealand, Spain, Sweden, United Kingdom and USA

† France, Germany, Italy, Japan, Spain, United Kingdom, & USA in phases 1 to 5; Australia and New Zealand, Belgium, Canada, and Sweden in phases 2 to 5; and the Gulf Cooperation Council (including Bahrain, Kuwait, Oman, Qatar, Saudi Arabia and United Arab Emirates), China, Russia, and Turkey in phase 5

§ mean value

¶ median at-risk time for the overall cohort

** median value

Table S4. Summary of baseline characteristics and main outcomes of identified studies (n=16) reporting mortality outcomes in the inter-dialytic period

Author, year, country (trial ID)	Study design	Population (N)	HD schedule	Inter-dialytic interval	Sudden death (%)	All-cause mortality		CV- mortality		CV events
						Timepoint	Value	Timepoint	Value	
Bleyer et al. 1999, USA (USRDS Case Mix Adequacy Study)	Retrospective, observational, multicentre	ESRD on HD (6,137)	MWF	Day after LIDI	20.8	NR	NR	NR	20.2%	NR
				Day after SIDI1	16.7	NR	NR	NR	14.9%	NR
				Day after SIDI2	14.2	NR	NR	NR	15.0%	NR
				End of LIDI	12.7	NR	NR	NR	13.3%	NR
			TTS	Day after LIDI	18.1	NR	NR	NR	18.5%	NR
				Day after SIDI1	13.5	NR	NR	NR	14.0%	NR
				Day after SIDI2	13.5	NR	NR	NR	13.5%	NR
				End of LIDI	13.7	NR	NR	NR	15.2%	NR
Bleyer et al. 2006, USA	Retrospective, observational, multicentre	ESRD on HD (80)	MWF or TTS	Day after LIDI (0-12h interval)	11.3	NR	NR	NR	NR	NR
				End of LIDI (60-72h interval)	20.6	NR	NR	NR	NR	NR
Foley et al. 2011, USA (ESRD Clinical Performance Measures Project)	Retrospective observational (cross-sectional), multicentre	ESRD on maintenance HD (32,065)	59.2% MWF 40.8% TTS	Day after LIDI	NR	1 year	22.1 per 100 person-years	1 year	10.2 per 100 person-years	Cardiac arrest per 100 person-years: 1.3 on day after LIDI vs. 1.0 on any other day MI per 100 person-years: 6.3 on day after LIDI vs. 4.4 on any other day
				Day after SIDI1			19.3 per 100 person-years		8.1 per 100 person-years	
				Day after SIDI2			18.3 per 100 person-years		7.9 per 100 person-years	
				End of LIDI			17.7 per 100 person-years		7.7 per 100 person-years	
Fotheringham et al. 2020, Europe*	Prospective observational (cohort), multicentre	CKD on maintenance HD (9,398)	MWF or TTS	Day after LIDI	NR	NR	8.3 per 100 person-years	NR	NR	NR
				Day after SIDI	NR	NR	4.3 per 100 person-years	NR	NR	NR
Fotheringham et al. 2019, Europe* (EURODOPPS)	Prospective observational (cohort), multicentre	CKD on maintenance HD (19,557)	55.5% MWF 44.7% TTS	Day after LIDI	NR	NR	17 per 100 person-years	NR	NR	NR
				Day after SIDI	NR	NR	14 per 100 person-years	NR	NR	NR
Genovesi et al. 2009, Italy	Retrospective, observational, multicentre	CKD on chronic HD (476)	MWF or TTS	During LIDI	40.6	NR	NR	NR	NR	NR
				During SIDI 1	34.4	NR	NR	NR	NR	NR
				During SIDI 2	25.0	NR	NR	NR	NR	NR
Karnik et al. 2001, USA	Prospective observational	ESRD on HD (400)	MWF	Day after LIDI	NR	NR	NR	NR	NR	Cardiac arrest %: 23.3
				Day after SIDI1	NR	NR	NR	NR	NR	Cardiac arrest %: 13.5

Author, year, country (trial ID)	Study design	Population (N)	HD schedule	Inter-dialytic interval	Sudden death (%)	All-cause mortality		CV- mortality		CV events
						Timepoint	Value	Timepoint	Value	
(FMCNA)	(cohort), single centre			Day after SIDI2	NR	NR	NR	NR	NR	Cardiac arrest %: 14.5
			TTS	Day after LIDI	NR	NR	NR	NR	NR	Cardiac arrest %: 16.3
				Day after SIDI1	NR	NR	NR	NR	NR	Cardiac arrest %: 14.3
				Day after SIDI2	NR	NR	NR	NR	NR	Cardiac arrest %: 17.0
Krishnasamy et al. 2013, Australia (ANZDATA)	Retrospective, observational (cohort), multicentre	ESRD on maintenance HD (10,338 patient deaths)	MWF or TTS	Day after LIDI - Mon	NR	NR	NR	NR	9.7%	MI (OR): 1.19
				Day after LIDI - Tue	NR	NR	NR	NR	8.8%	MI (OR): 1.13
				Day after SIDI1 - Wed	NR	NR	NR	NR	8.0%	MI (OR): 1.02
				Day after SIDI1 - Thurs	NR	NR	NR	NR	8.0%	MI (OR): 1.05
				Day after SIDI2 - Fri	NR	NR	NR	NR	7.7%	MI (OR): 1.06
				Day after SIDI2 - Sat	NR	NR	NR	NR	7.6%	MI (OR): 0.86
				End of LIDI - Sun	NR	NR	NR	NR	6.7%	MI (OR): 0.76
Kumar et al. 2013, India	Prospective observational	ESRD on maintenance HD (240)	MT or TF or WS	Day after LIDI	NR	NR	NR	2 years	12.9%	NR
				Day after SIDI	NR	NR	NR	2 years	6.3%	NR
Lafrance, et al. 2006, Canada	Retrospective, observational, single centre	CKD on HD (257)	MWF or TTS	Day after LIDI	NR	NR	NR	NR	NR	CPR 52.6%
				Day after SIDI1	NR	NR	NR	NR	NR	CPR 21.1%
				Day after SIDI2	NR	NR	NR	NR	NR	CPR 26.3%
Rhee et al. 2017, USA	Prospective, observational, multicentre	CKD on maintenance HD (MWF: n=61,152; TTS: n=43,968)	MWF	Day after LIDI	NR	NR	23.7%	NR	NR	NR
				Day after SIDI1	NR	NR	14.7%	NR	NR	NR
				Day after SIDI2	NR	NR	15.4%	NR	NR	NR
				End of LIDI	NR	NR	15.4%	NR	NR	NR
			TTS	Day after LIDI	NR	NR	22.3%	NR	NR	NR
				Day after SIDI1	NR	NR	16.2%	NR	NR	NR
				Day after SIDI2	NR	NR	15.9%	NR	NR	NR
				End of LIDI	NR	NR	15.1%	NR	NR	NR
Roy-Chaudhury et al. 2018, USA and India	Prospective, multicentre,	ESRD on HD (66)	MWF or TTS	Day after LIDI	NR	NR	NR	NR	NR	Arrhythmia: 0.0023 rate per hour; AF: 0.00019 rate per hour

Author, year, country (trial ID)	Study design	Population (N)	HD schedule	Inter-dialytic interval	Sudden death (%)	All-cause mortality		CV- mortality		CV events
						Timepoint	Value	Timepoint	Value	
(MiD, NCT01779856)	single group, open-label trial			Day after SIDI1	NR	NR	NR	NR	NR	Arrhythmia: 0.0004 rate per hour; AF: 0.00024 rate per hour
				Day after SIDI2	NR	NR	NR	NR	NR	Arrhythmia: 0.0002 rate per hour; AF: 0.00018 rate per hour
				End of LIDI (60-72h interval)	NR	NR	NR	NR	NR	Arrhythmia: 0.0012 rate per hour; AF: 0.00014 rate per hour
				End of SIDI1 (36-48h interval)	NR	NR	NR	NR	NR	Arrhythmia: 0.0008 rate per hour; AF: 0.00013 rate per hour
				End of SIDI2 (36-48h interval)	NR	NR	NR	NR	NR	Arrhythmia: 0.0008 rate per hour; AF: 0.0008 rate per hour
Tangweerapong et al. 2017, Thailand	Prospective, observational, single centre	ESRD on chronic HD (28)	MWF or TTS	During LIDI	NR	NR	NR	NR	NR	Cardiac Arrhythmia 28.6%; Ventricular arrhythmia 10.7%
Tumlin et al. 2019, USA and India (MiD, NCT01779856)	Prospective, multicentre, single group, open-label trial	ESRD on HD (66)	MWF or TTS	Day after LIDI	NR	NR	NR	NR	NR	Arrhythmia: 0.014 rate per hour
				End of LIDI (60-72h interval)	NR	NR	NR	NR	NR	Arrhythmia: 0.019 rate per hour
				Day after SIDI1	NR	NR	NR	NR	NR	Arrhythmia: 0.012 rate per hour
				End of SIDI1 (36-48h interval)	NR	NR	NR	NR	NR	Arrhythmia: 0.017 rate per hour
				Day after SIDI2	NR	NR	NR	NR	NR	Arrhythmia: 0.014 rate per hour
				End of SIDI2 (36-48h interval)	NR	NR	NR	NR	NR	Arrhythmia: 0.021 rate per hour
Wong et al. 2015, Australia (ACTRN12613001326785)	Prospective observational (cohort), multicentre	CKD on HD (50)	MWF or TTS	End of LIDI	NR	NR	NR	NR	NR	Arrhythmia 16.6%
				Day after LIDI	NR	NR	NR	NR	NR	Arrhythmia 36.0%
				Day after SIDI1	NR	NR	NR	NR	NR	Arrhythmia 13.6%
				Day after SIDI2	NR	NR	NR	NR	NR	Arrhythmia 15.2%
Zhang et al. 2012, International[†] (DOPPS)	Prospective, observational (cohort), multicentre	CKD on maintenance HD	MWF	Day after LIDI (USA)	NR	NR	19.7%	NR	RR: 1.45	NR
				Day after SIDI1 (USA)	NR	NR	14.8%	NR	RR: 1.06	NR

Author, year, country (trial ID)	Study design	Population (N)	HD schedule	Inter-dialytic interval	Sudden death (%)	All-cause mortality		CV- mortality		CV events
						Timepoint	Value	Timepoint	Value	
		(USA: n=4,666; Europe: n=5,623; Japan: n=3,531)		Day after SIDI2 (USA)	NR	NR	14.6%	NR	RR: 1.05	NR
				End of LIDI (USA)	NR	NR	12.4%	NR	RR: 0.94	NR
				Day after LIDI (Europe)	NR	NR	19.6%	NR	RR: 1.55	NR
				Day after SIDI1 (Europe)	NR	NR	14.8%	NR	RR: 0.87	NR
				Day after SIDI2 (Europe)	NR	NR	14.2%	NR	RR: 1.33	NR
				End of LIDI (Europe)	NR	NR	12.4%	NR	RR: 0.89	NR
				Day after LIDI (Japan)	NR	NR	17.9%	NR	RR: 1.62	NR
				Day after SIDI1 (Japan)	NR	NR	13.3%	NR	RR: 0.84	NR
				Day after SIDI2 (Japan)	NR	NR	19.1%	NR	RR: 0.75	NR
				End of LIDI (Japan)	NR	NR	12.1%	NR	RR: 1.12	NR
			TTS	Day after LIDI (USA)	NR	NR	19.2%	NR	RR: 1.56	NR
				Day after SIDI1 (USA)	NR	NR	14.2%	NR	RR: 1.03	NR
				Day after SIDI2 (USA)	NR	NR	14.6%	NR	RR: 1.09	NR
				End of LIDI (USA)	NR	NR	15.7%	NR	RR: 1.16	NR
				Day after LIDI (Europe)	NR	NR	16.9%	NR	RR: 1.43	NR
				Day after SIDI1 (Europe)	NR	NR	11.9%	NR	RR: 0.75	NR
				Day after SIDI2 (Europe)	NR	NR	18.0%	NR	RR: 1.88	NR
				End of LIDI (Europe)	NR	NR	13.6%	NR	RR: 0.85	NR
				Day after LIDI (Japan)	NR	NR	19.1%	NR	RR: 0.75	NR

Author, year, country (trial ID)	Study design	Population (N)	HD schedule	Inter-dialytic interval	Sudden death (%)	All-cause mortality		CV- mortality		CV events
						Timepoint	Value	Timepoint	Value	
				Day after SIDI1 (Japan)	NR	NR	15.5%	NR	RR: 1.00	NR
				Day after SIDI2 (Japan)	NR	NR	17.9%	NR	RR: 1.93	NR
				End of LIDI (Japan)	NR	NR	10.7%	NR	RR: 0.98	NR

AF: atrial fibrillation; CKD: chronic kidney disease; CPR: cardiopulmonary resuscitation; ESDR: end-stage renal disease; HD: haemodialysis; LIDI: long inter-dialytic interval; MI: myocardial infarction; MT: Monday, Thursday; MWF: Monday, Wednesday, Friday; NR: not reported; OR: odds ratio; RR: relative risk; SIDI: short inter-dialytic interval; TF: Tuesday, Friday; TTS: Tuesday, Thursday, Saturday; WS: Wednesday, Saturday

* Belgium, France, Germany, Italy, Sweden, Spain, and United Kingdom

† USA, Japan, Belgium, France, Germany, Italy, Spain, Sweden, and United Kingdom
